# 2025 Thai guideline for the diagnosis and management of atrial fibrillation

**DOI:** 10.2478/abm-2025-0028

**Published:** 2025-10-31

**Authors:** Tachapong Ngarmukos, Komsing Methavigul, Voravut Rungpradubvong, Sirin Apiyasawat, Wanwarang Wongcharoen, Satchana Pumprueg, Warangkana Boonyapisit, Arisara Suwannagool, Thoranis Chantrarat, Pattarapong Makarawate, Treechada Wisaratapong, Kumpol Chintanavilas, Panyapat Jiampo, Rungroj Krittayaphong

**Affiliations:** 1 Department of Medicine, Faculty of Medicine, Ramathibodi Hospital, Mahidol University, Bangkok 10400, Thailand; 2 Department of Cardiology Central Chest Institute of Thailand, Nonthaburi 11000, Thailand; 3 Department of Medicine, Faculty of Medicine, Chulalongkorn University, Bangkok 10330, Thailand; 4 Department of Medicine, Faculty of Medicine, Chiang Mai University, Chiang Mai 50200, Thailand; 5 Department of Medicine, Faculty of Medicine, Siriraj Hospital, Mahidol University, Bangkok 10700, Thailand; 6 Department of Medicine, Phramongkutklao College of Medicine, Bangkok 10400, Thailand; 7 Department of Medicine, Faculty of Medicine, Khon Kaen University, Khon Kaen 40002, Thailand; 8 Department of Medicine, Faculty of Medicine, Prince of Songkla University, Songkla 90110, Thailand; 9 Department of Medicine, Faculty of Medicine, Thammasat University, Rangsit Campus, Prathumthani 12120, Thailand; 10 Department of Medicine, Bhumibol Adulyadej Hospital, Bangkok 10220, Thailand

**Keywords:** atrial fibrillation, device, ischemic stroke, major bleeding, mortality

## Abstract

**Background:**

The Thai Cardiac Electrophysiology Club, part of the Heart Association of Thailand under the Royal Patronage of His Majesty the King, published a Clinical Practice Guideline for the Management of Patients with Atrial Fibrillation (AF) in 2012. The availability of new evidence indicates a significant need for the updating of the existing clinical practice guideline.

**Objective:**

To serve as guidelines on the diagnosis and management of Thai patients with AF.

**Methods:**

Meetings were conducted from September 2024 to March 2025, including a public hearing that engaged various stakeholders. The final Thai version received approval in February 2025, while the English translation was completed in April 2025.

**Results:**

AF is highly prevalent. The diagnosis is crucial to detect those who need treatment for the prevention of complication. Holistic management focused on stroke prevention, symptom management, and management of cardiovascular risk factors; and comorbidity is the key success factor to maintain a good quality of life. Emerging evidence regarding newer classes of anticoagulant indicates that these treatments are effective and safe for stroke prevention. Newer catheter ablation technology has been developed and provides a better success rate and lower rate of complication. The newer stroke risk prediction CHA_2_DS_2_-VA Score is recommended for the Thai population due to its simplicity.

**Conclusion:**

The 2025 updated AF clinical practice guidelines establish a framework, provide recommendations, and serve as a comprehensive resource for the contemporary diagnosis and management of AF in the Thai population, with the goal of preventing complications.

## Abbreviations

**Table:** 

5-FU	5-fluorouracil	ICD	Implantable cardioverter-defibrillator
ACEi	Angiotensin receptor enzyme inhibitor	ILR	Implantable loop recorder
ACS	Acute coronary syndromes	INR	International normalized ratio
AF	Atrial fibrillation	LA	Left atrium
AFL	Atrial flutter	LAA	Left atrial appendage
AHRE	Atrial high-rate episode	LAAO	Left atrial appendage occlusion
AI	Artificial intelligence	LMWH	Low molecular weight heparin
ARB	Angiotensin receptor blocker	LVEF	Left ventricular ejection fraction
AT	Atrial tachycardia	METs	Metabolic equivalents
AV	Atrioventricular	Mg	Magnesium
AVN	Atrioventricular node	MI	Myocardial infarction
AVRT	Atrioventricular re-entrant	MRA	Mineralocorticoid receptor antagonists
BCR-ABL	Breakpoint cluster region-Abelson oncogene locus	MS	Mitral stenosis
BMI	Body mass index	NDCCB	Non-dihydropyridine calcium-channel blocker
BNP	B-type natriuretic peptide	NSAIDs	Non-steroidal anti-inflammatory drugs
BTK	Bruton tyrosine kinase	NSTE-ACS	Non-ST elevation acute coronary syndrome
CABG	Coronary artery bypass grafting	NT-proBNP	N-terminal pro-B-type natriuretic peptide
CAD	Coronary artery disease	NYHA	New York Heart Association
CAR-T	Chimeric antigen receptor T cell	OAC	Oral anticoagulant
CBC	Complete blood count	OSA	Obstructive sleep apnea
CCS	Chronic coronary syndromes	P2Y_12_i	P2Y_12_-receptor inhibitor
CIED	Cardiac implantable electronic device	PAD	Peripheral arterial disease
CKD	Chronic kidney disease	PCC	Prothrombin complex concentrate
CMR	Cardiac magnetic resonance	PCI	Percutaneous coronary intervention
COPD	Chronic obstructive pulmonary disease	POAF	Post-operative atrial fibrillation
CPAP	Continuous positive airway pressure	PPG	Photoplethysmography
CrCl	Creatinine clearance	PPI	Proton pump inhibitor
CRT	Cardiac resynchronization therapy	PVI	Pulmonary vein isolation
CSP	Conduction system pacing	RCT	Randomized controlled trial
CT	Computed tomography	RHD	Rheumatic heart disease
CTA	Computed tomography angiography	SBP	Systolic blood pressure
CYP	Cytochrome P450	SGLT2i	Sodium-glucose cotransporter-2 inhibitor
DBP	Diastolic blood pressure	STEMI	ST-elevation myocardial infarction
DOAC	Direct oral anticoagulant	TAVI	Trans-aortic valve intervention
ECG	Electrocardiogram	TEE	Transesophageal echocardiography
EGM	Electrogram	TIA	Transient ischemic attack
FFP	Fresh frozen plasma	TKIs	Tyrosine kinase inhibitors
GDMT	Guideline-directed management and therapy	TTE	Transthoracic echocardiography
GI	Gastrointestinal	TTR	Time in therapeutic range
HbA1c	Hemoglobin A1c	UFH	Unfractionated heparin
HCM	Hypertrophic cardiomyopathy	VEGF	Vascular endothelial growth factor
HF	Heart failure	VF	Ventricular fibrillation
HFpEF	Heart failure with preserved ejection fraction	VKA	Vitamin K antagonist
HFrEF	Heart failure with reduced ejection fraction	VKORC1	Vitamin K epoxide reductase complex subunit 1
HTN	Hypertension	WPW	Wolff-Parkinson-White

## Acronyms

**Table:** 

AFFIRM	Atrial Fibrillation Follow-up Investigation of Rhythm Management	EAST-AFNET 4	Early Treatment of Atrial Fibrillation for Stroke Prevention Trial-Atrial Fibrillation Network 4
APAF-CRT	Ablate and Pace for Atrial Fibrillation and Cardiac Resynchronization Therapy	HOT CAFE	How to Treat Chronic Atrial Fibrillation
CASTLE-AF	Catheter Ablation versus Standard Conventional Therapy in Patients with Left Ventricular Dysfunction and Atrial Fibrillation	ORBIT-AF	Outcomes Registry for Better Informed Treatment of Atrial Fibrillation
CASTLE-HTx	Catheter Ablation for Atrial Fibrillation in Patients with End-stage Heart Failure and Eligibility for Heart Transplantation	PALACS	Effect of Posterior Pericardiotomy on the Incidence of Atrial Fibrillation After Cardiac Surgery
COOL AF Thailand	A Cohort of Antithrombotic Use and Optimal INR Level in Patients with Non-valvular Atrial Fibrillation in Thailand	PRAGUE-17	Left Atrial Appendage Closure vs. Novel Anticoagulation Agents in Atrial Fibrillation
		RACE II	Rate Control Efficacy in Permanent Atrial Fibrillation: A Comparison Between Lenient Versus Strict Rate Control II

## Preamble

The Thai guideline for the diagnosis and management of atrial fibrillation (AF) was prepared by the Thai Cardiac Electrophysiology Club, part of the Heart Association of Thailand under the Royal Patronage of His Majesty the King. This clinical practice guideline is based on scientific evidence and expert recommendations regarding the management of AF. It serves as a guideline for physicians and healthcare professionals in Thailand, adapted to the local healthcare context to enhance the standard of AF care. The implementation of this guideline aims to reduce mortality rates, hospital admissions, and disability associated with AF.

However, this clinical practice guideline provides recommendations based on the best available scientific evidence at the time of publication and may be adapted as appropriate, depending on the physician’s judgment. The application of these recommendations should consider the diversity of clinical presentations, patient-specific factors, healthcare facility capabilities, availability of medical equipment, personnel readiness, and laboratory testing capabilities. Moreover, patient safety, treatment efficacy, cost-effectiveness, and quality of life should always be taken into account. This guideline is not intended to be rigid regulations that must be followed in every case, and it may not cover all specific clinical scenarios. Ultimately, decisions should be made collaboratively between physicians and patients. Due to the large amount of content, the Thai AF Guideline is presented in 2 parts which cover 8 topics: (1) definitions, diagnosis, and screening; (2) patient assessment upon diagnosis of AF; (3) stroke prevention in AF patients; (4) heart rate control with medications; (5) management to maintain sinus rhythm with medications and cardioversion; (6) catheter ablation; (7) management od comorbidities; and (8) treatment of AF in specific populations.

## Class of recommendation

The class of recommendation is established to indicate the extent to which a given recommendation in this clinical practice guideline is expected to benefit or harm patients.

**Table:** 

**I**	“Is recommended” There is clear and consistent scientific evidence or expert consensus that the intervention is beneficial and cost-effective for patients.
**IIa**	“Should be considered” Scientific evidence or expert opinion is somewhat conflicting, but the overall consensus suggests that the intervention is likely beneficial and cost-effective.
**IIb**	“May be considered” Scientific evidence or expert opinion is somewhat conflicting, and the overall consensus suggests potential benefit, though the certainty of benefit is less clear.
**III**	“Is not recommended” There is clear and consistent scientific evidence or expert consensus that the intervention is not beneficial or may be harmful in certain cases.

## Level of evidence

The level of evidence is established to indicate the reliability of the data supporting the recommendations in this guideline.

**Table:** 

**A**	Evidence derived from multiple randomized clinical trials or meta-analyses.
**B**	Evidence derived from a single randomized clinical trial or large non-randomized studies.
**C**	Evidence derived from small studies, retrospective studies, registries, or expert opinion.

## Definitions, diagnosis, and screening

### Definitions

1. AF is an arrhythmia caused by an abnormal electrical circuit in the atria, resulting in an irregular and ineffective contraction. Diagnosis of AF requires an electrocardiogram (ECG). Patients with AF may also present with other types of atrial arrhythmias, particularly atrial flutter (AFL) and atrial tachycardia (AT).
2. Clinical AF refers to AF detected using a standard 12-lead ECG (typically lasting about 10 s) or other screening methods such as ambulatory ECG monitoring, where an episode lasting 30 s or more is considered significant, regardless of whether the patient exhibits symptoms.
3. AFL is an arrhythmia caused by an abnormal electrical circuit in the atria, similar to AF, but occurring at a fixed location, leading to a regularized ECG pattern with flutter waves. In untreated patients, these flutter waves typically appear at a rate of 240–320 beats/min (bpm).
4. AT is a rapid atrial arrhythmia originating from an abnormal electrical focus within the atria. The ECG shows P waves that do not originate from the sinus node. AT can be distinguished from AFL by observing the isoelectric line between P waves.
5. Device-detected subclinical AF refers to AF detected in asymptomatic patients who have never had a 12-lead ECG showing AF, but were diagnosed using an intracardiac electrogram (EGM) recorded by a cardiac implantable electronic device (CIED).
6. Atrial high-rate episode (AHRE) refers to abnormally fast atrial electrical signals detected and recorded by a CIED. AHRE may be caused by AF, other arrhythmias, or noise interference. A confirmed diagnosis of AF requires further verification through intracardiac EGM stored in the CIED’s memory.
7. Oral anticoagulants (OACs) refer to blood-thinning medications taken orally, such as warfarin and direct oral anticoagulants (DOACs).
8. Antiplatelets refer to medications that prevent platelet aggregation, such as aspirin or clopidogrel.
9. Fibrinolytic agents refer to medications that dissolve blood clots, such as streptokinase.
10. Rate control refers to treatment aimed at controlling heart rate.
11. Rhythm control refers to treatment aimed at restoring normal heart rhythm.

### Classification of AF

1. First-diagnosed AF refers to AF diagnosed for the first time, without classification as paroxysmal, persistent, long-standing persistent, or permanent AF.
2. Paroxysmal AF refers to AF episodes lasting no more than 7 days, which may terminate spontaneously or require treatment.
3. Persistent AF refers to AF episodes lasting for more than 7 days.
4. Long-standing persistent AF refers to AF persisting for more than 12 months, but treatment remains focused on restoring normal heart rhythm (rhythm control).
5. Permanent AF refers to continuous AF, where both the patient and healthcare provider agree that rate control is the primary focus rather than rhythm restoration.

### Diagnosis of AF

AF is a type of arrhythmia that can be diagnosed using a multiple-lead or single-lead ECG recording lasting more than 30 s, or a 12-lead ECG [1, 2]. The characteristic findings include the absence of distinct P waves and an irregularly irregular ventricular rhythm. The QRS complexes are typically irregular (Table 1).

**Table 1.** Recommendations for diagnosing AF

Recommendation	Class^a^	Evidence^b^
It is recommended to use a 12-lead ECG, multiple-lead ECG, or singlelead ECG to confirm the diagnosis of clinical AF and initiate risk assessment and treatment.	I	B

1 AF, atrial fibrillation; ECG, electrocardiogram.

a Class of recommendation.

b Level of evidence.

### Screening for AF patients

Screening for AF can be conducted through opportunistic or systematic screening. Opportunistic screening involves detecting AF when patients visit healthcare facilities for other medical conditions, such as during routine check-ups or annual vaccinations. Systematic screening involves actively inviting a specific population group, such as all individuals over the age of 65 years in a designated area, to undergo AF screening. Previous studies have shown no significant difference in the number of AF cases detected between opportunistic and systematic screening [3]. However, the key consideration is ensuring a clear and well-defined management plan for patients identified through the screening process.

A systematic screening study for AF was conducted in Phetchaburi and Lopburi provinces [4] using blood pressure monitors equipped with pulse irregularity detection to screen a total of 13,864 individuals aged ≥65 years. The study found an AF prevalence of 2.8% (393 individuals). However, only 58% (226 individuals) of those screened sought further evaluation at a hospital, and among those who underwent ECG testing, 33% (75 individuals) were confirmed to have AF. Additionally, another systematic screening study for AF was conducted in the Mae Rim district, Chiang Mai province, using a single snapshot 12-lead ECG to screen 1,277 individuals aged ≥65 years. The study found an AF prevalence of 1.9% (24 individuals) [5].

Pulse palpation is the simplest method for AF screening, with a sensitivity of 87%–97% and a specificity of 70%–80%. However, studies indicate that the rate of physician compliance with pulse palpation for AF screening remains low [2].

Hypertension (HTN) and AF commonly coexist, and HTN further increases the risk of stroke in AF patients. Currently, automated blood pressure monitors equipped with pulse irregularity detection programs are available for AF screening, with a sensitivity of 93%–100% and a specificity of 86%–92% [6].

**Figure 1.**
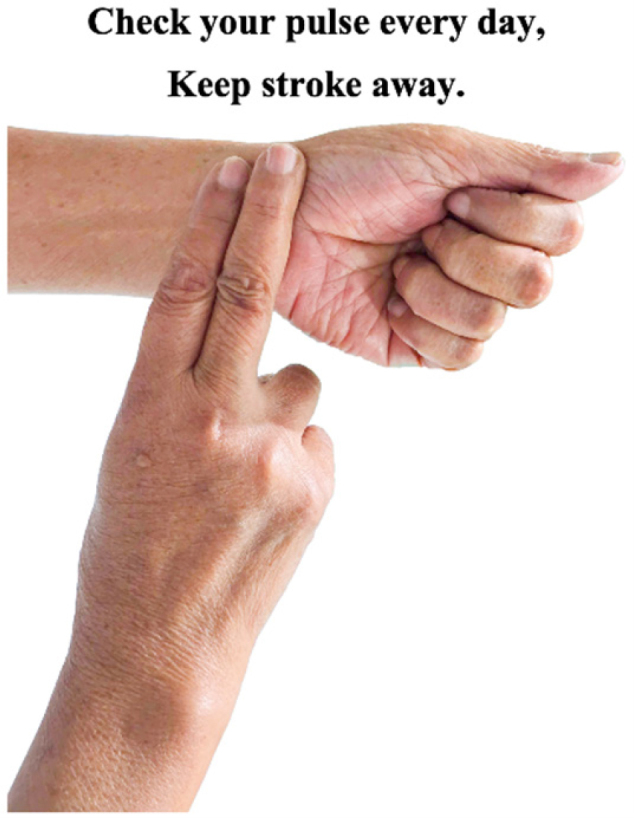
Illustration of the pulse palpation method for AF screening.

AF detected through pulse palpation or an automated blood pressure monitor must always be confirmed with an ECG to establish a definitive diagnosis (Table 2 and Figure 1).

**Table 2.** Recommendations for AF screening

Recommendation	Class^a^	Evidence^b^
Opportunistic screening for AF is recommended using pulse palpation or a blood pressure monitor capable of detecting pulse irregularity in individuals aged ≥65 years, particularly during medical visits for other conditions. If abnormalities are detected through pulse palpation or blood pressure measurement, an ECG should be performed to confirm the diagnosis.	I	C

1 AF, atrial fibrillation; ECG, electrocardiogram.

a Class of recommendation.

b Level of evidence.

According to the 2024 ESC Guidelines for the management of AF, developed in collaboration with the European Association for Cardio-Thoracic Surgery, population-based screening for AF using a prolonged non-invasive ECG-based approach should be considered in individuals aged ≥75 years, or ≥65 years with additional CHA_2_DS_2_-VA risk factors, to ensure earlier detection of AF (Class IIa recommendation, Level of Evidence B) [2]. However, in Thailand, due to limited resources, we recommend opportunistic screening for AF using pulse palpation or a blood pressure monitor capable of detecting pulse irregularity in individuals aged ≥65 years.

The detection of AF in patients with embolic stroke of undetermined source (ESUS) increases with longer durations of cardiac monitoring [7], ranging from 2% with 1 week of monitoring to >20% by 3 years [8]. In patients with ESUS who have factors associated with an increased detection of AF including age increase, left atrial enlargement, cortical location of stroke, and an increased number of PACs per 24 h [9, 10] prolonged monitoring is recommended.

### Tools and devices for AF screening and diagnosis

Devices used for AF screening and diagnosis can be classified into two main categories:

1. ECG-based devices for AF screening and diagnosis:
  1.1 12-lead ECG [3]—The standard method for detecting AF.
  1.2 Continuously recording or loop recording ECG devices—These use electrode patches attached to the body, such as: Holter monitoring, which provides continuous ECG recording for 24–48 h [11]; Wearable ECG patches, capable of recording for 14–28 days [12–14]; and Mobile cardiac telemetry, used for monitoring ECG in hospitalized patients [15].
  1.3 Implantable cardiac monitors [implantable loop recorders (ILRs)]—These devices provide continuous ECG recording with a battery life of 3–5 years [16, 17].
  1.4 CIEDs—Includes pacemakers and implantable cardioverter-defibrillators (ICDs), which can detect and manage arrhythmias.
  1.5 Handheld ECG devices and smartwatches—Portable ECG devices that can record ECG continuously for at least 30 s [18]. Some smartwatches have built-in ECG recording capabilities for at least 30 s [19, 20].
2. Devices that use methods other than ECG for AF screening, such as photoplethysmography (PPG) and artificial intelligence (AI) technology.

PPG is a technology used to detect changes in blood circulation within the body by measuring variations in blood volume passing through the capillaries in the skin. This is achieved using smartphone and smartwatch cameras that emit infrared light through the skin. The collected data is then processed through AI algorithms to generate signals that can be analyzed for heart rate assessment or various health conditions, such as AF, respiration, and blood circulation. Although these devices can screen for AF, it is still recommended to perform an ECG, either single-lead or multiple-lead, recorded for >30 s, or a standard 12-lead ECG to confirm the diagnosis of AF. While AI has become increasingly significant in AF screening and diagnosis, enhancing accuracy and speed, its widespread adoption still requires further studies on efficacy, accuracy, and ethical considerations.

In summary, the diagnosis of AF still requires clear and accurate ECG data. Despite the development of numerous new devices, ECG data remains essential for enhancing diagnostic accuracy and ensuring appropriate patient management.

### Patient assessment upon diagnosis of AF

Patients diagnosed with AF should undergo further diagnostic evaluation and a comprehensive medical history review to identify risk factors and/or comorbid conditions that require concurrent management. Recommendations for additional diagnostic evaluations in newly diagnosed AF patients are presented in Table 3, while detailed assessment criteria are outlined in Table 4.

**Table 3.** Recommendations for additional diagnostic evaluation in newly diagnosed AF patients

Recommendation	Class^a^	Evidence^b^
It is recommended that newly diagnosed AF patients undergo additional diagnostic evaluation to aid in treatment planning, as follows: TTE to assess cardiac structure [21–23]. Basic laboratory tests, including CBC, blood glucose levels, serum electrolytes, liver function tests, renal function tests, and thyroid function tests. Screening for comorbid conditions to evaluate risk factors for thromboembolism and bleeding.	I	A

1 AF, atrial fibrillation; CBC, complete blood count; TTE, transthoracic echocardiography.

a Class of recommendation.

b Level of evidence.

**Table 4.** Detailed assessment and additional diagnostic evaluations in newly diagnosed AF patients

Newly diagnosed patients	Newly diagnosed patients (selected cases)
1. Symptom and complication assessment	
2. Comprehensive medical history review to identify	
2.1. AF pattern	
2.2. Comorbidities	
2.3. Family history	
2.4. Current medications	
2.5. Risk factors for thromboembolism and bleeding	
3. 12-lead ECG for assessing	1. Ambulatory ECG monitoring for assessing the relationship between AF and symptoms, as well as treatment response
3.1. Confirmation of AF diagnosis	
3.2. Heart rate	1.1. AF burden (frequency and duration of AF episodes).
3.3. Pre-existing structural heart disease, conduction abnormalities, or myocardial ischemia	1.2. Ventricular rate control
	2. Exercise ECG for assessing the effects of antiarrhythmic drugs and/or myocardial ischemia.
4. Blood tests to identify comorbidities or conditions that may trigger AF or increase the risk of bleeding or thromboembolism	3. Additional blood tests for investigation of cardiovascular conditions
	3.1. NT-proBNP
4.1. CBC	3.2. Troponin
4.2. Coagulogram	
4.3. Kidney function tests	
4.4. Serum electrolytes	
4.5. Liver function tests	
4.6. Blood glucose or HbA1c levels	
4.7. Thyroid function tests	
5. TTE for assessing	4. TEE for assessing
5.1. Left atrial size and function	4.1. Left atrial thrombus evaluation
5.2. Valve function	4.2. Valvular disease assessment
5.3 LVEF	5. Radiological imaging assessments
	5.1. Chest X-ray to assess AF-related complications and associated comorbidities
	5.2. Coronary CTA in patients with suspected CAD
	5.3. CMR to evaluate atrial and ventricular cardiomyopathies and assist in procedural planning
	5.4. Brain imaging and cognitive function assessment to evaluate cerebrovascular disease and risk of dementia

1 AF, atrial fibrillation; CAD, coronary artery disease; CBC, complete blood count; CMR, cardiac magnetic resonance; CTA, computed tomography angiography; ECG, electrocardiogram; HbA1c, hemoglobin A1c; LA, left atrium; LVEF, left ventricular ejection fraction; NT-proBNP, N-terminal pro-B-type natriuretic peptide; TEE, transesophageal echocardiography; TTE, transthoracic echocardiography.

### AF patient management guidelines

**Figure 2.**
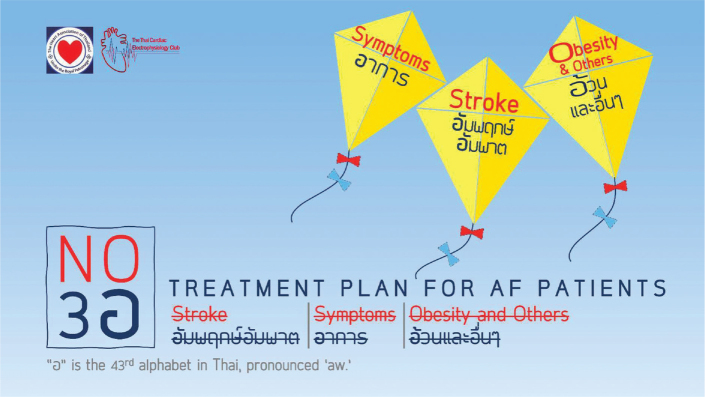
Illustration of the management guidelines for AF patients. AF, atrial fibrillation.

Patients diagnosed with AF (Figure 2) should have a structured treatment plan following the **“No 3 อ”** principle, which includes:

No อัมพฤกษ์-อัมพาต—Prevention of stroke
No อาการ—Management of symptoms
No อ้วนและอื่นๆ—Treatment of obesity and comorbidities

The treatment approach based on the holistic integration principle, known as the “No 3 อ” has been studied and shown to be effective in AF patients in various countries, including some in Asia. This principle is also recognized internationally by the acronym ABC (Atrial Fibrillation Better Care) (A = Avoid stroke, B = Better symptom management, C = Comorbidity and cardiovascular risk factors management). Patients with ABC compliant have been shown in the COOL-AF registry to have a better clinical outcome compared to those with ABC not compliant [24, 25]. While the terminology differs, the underlying concept remains the same as the “No 3 อ” strategy outlined in this clinical practice guideline.

A systematic review and meta-analysis found that, compared to standard AF management, patients treated using this approach had a 58% reduction in overall mortality risk, a 63% reduction in cardiovascular-related mortality risk, a 45% reduction in stroke incidence, and a 31% reduction in major bleeding complications. Therefore, a collaborative effort among physicians and other healthcare professionals to manage AF patients using the “No 3 อ” approach will benefit both patients and their families, helping to preserve quality of life and reduce the financial burden of stroke care [26].

### No อัมพฤกษ์-อัมพาต—Prevention of stroke

Consider stroke prevention in patients at risk of thromboembolism by appropriately using OACs while minimizing the risk of bleeding complications associated with anticoagulant therapy.

### No อาการ—Management of symptoms

Reduce symptoms and AF-related morbidity through effective rate and rhythm control strategies to decrease hospitalizations and improve prognosis. Rhythm control should be considered when beneficial, while carefully assessing the associated risks for each patient.

### No อ้วนและอื่นๆ—Treatment of obesity and comorbidities

Screen for comorbid conditions, including obesity, diabetes, HTN, heart failure (HF), and obstructive sleep apnea (OSA), as well as modifiable risk factors such as physical inactivity and alcohol consumption. It is crucial to guide all AF patients on managing comorbidities and avoiding risk factors to improve overall health outcomes.

### Stroke prevention in AF patients

AF significantly raises the risk of thromboembolism and ischemic stroke [27]. Strategies for stroke prevention can be classified by treatment approaches and risk levels as follows:

### Initial considerations for the use of OACs

**Figure 3.**
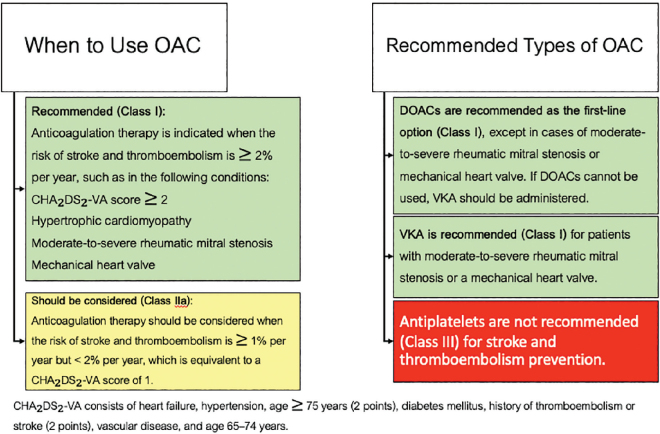
Guidelines for selecting OACs. DOAC, direct oral anticoagulant; OAC, oral anticoagulant; VKA, vitamin K antagonist.

It is recommended that all AF patients be considered for OAC therapy, except in cases where contraindications exist or the patient is at low risk for stroke and thromboembolism, with an estimated annual risk of <1% (Figure 3).

There are two types of OACs: vitamin K antagonists (VKAs) and DOACs. There is strong scientific evidence supporting the effectiveness of OACs in reducing ischemic stroke and thromboembolism risk, with additional data confirming their efficacy specifically in Asian populations [28].

On the contrary, antiplatelet agents have been shown to have low efficacy and an increased risk of bleeding complications [28]. Therefore, the use of antiplatelet therapy alone is not recommended for the prevention of ischemic stroke and thromboembolism in AF patients.

## Thromboembolic risk assessment

### Risk stratification

The risk of ischemic stroke and thromboembolism can be categorized into three levels (Table 5):

**Table 5.** Recommendations for assessing the risk of ischemic stroke and thromboembolism in AF patients

Recommendation	Class^a^	Evidence^b^
OAC therapy is recommended for AF patients with a risk of ischemic stroke and thromboembolism ≥2%/year, including patients with a CHA_2_DS_2_-VA score ≥2 [29, 30].	I	A
Regular risk assessment for ischemic stroke and thromboembolism in AF patients is recommended [31].	I	B
OAC therapy should be considered for AF patients with a risk of ischemic stroke and thromboembolism ≥1% but <2%/year, including those with a CHA_2_DS_2_-VA score of 1, while carefully weighing the benefits of stroke prevention against the individual bleeding risk [30].	IIa	C
Antiplatelet therapy is not recommended for preventing ischemic stroke and thromboembolism in AF patients [28].	III	A

1 AF, atrial fibrillation; OAC, oral anticoagulant.

a Class of recommendation.

b Level of evidence.

#### High risk

Patients with an annual risk of ≥2% for ischemic stroke and thromboembolism are recommended to receive OAC therapy [29].

#### Moderate risk

Patients with an annual risk of ≥1% but <2% for ischemic stroke and thromboembolism should be considered for OAC, balancing the benefits of stroke and thromboembolism risk reduction versus the individual patient’s bleeding risk [29, 30].

#### Low risk

Patients with an annual risk of <1% for ischemic stroke and thromboembolism do not require OAC therapy [29, 30].

### Risk assessment methods

**Figure 4.**
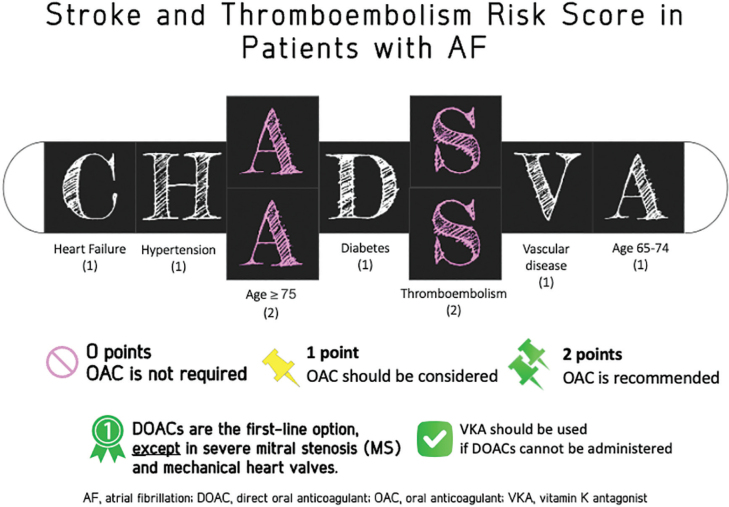
Utilization of CHA_2_DS_2_-VA score for assessing the risk of ischemic stroke and thromboembolism. AF, atrial fibrillation; DOAC, direct oral anticoagulant; OAC, oral anticoagulant; VKA, vitamin K antagonist.

For patients with AF, the CHA_2_DS_2_-VA risk scoring system is recommended. This system assigns points based on HF, HTN, age (2 points for those ≥75 years), diabetes, history of thromboembolism or ischemic stroke (2 points), vascular disease, and age 65–74 years [32]. Unlike the CHA_2_DS_2_-VASc score, CHA_2_DS_2_-VA omits sex as a risk factor (Table 6 and Figure 4), simplifying decisions regarding OAC prescription. Comparative studies between the two systems have shown that CHA_2_DS_2_-VA performs as well as or better than CHA_2_DS_2_-VASc in assessing ischemic stroke and thromboembolism risk [33]. CHA_2_DS_2_-VA score has been validated in the Thai population in the COOL-AF registry [34].

**Table 6.** Definitions of CHA_2_DS_2_-VA risk score components based on the definitions used in CHA_2_DS_2_-VASc score studies [1, 2, 32]

Risk factor	Definition	Score
C (congestive HF)	Symptoms and signs of right, left, or biventricular HF, confirmed by objective evidence of cardiac dysfunction through either non-invasive or invasive measurements, or LVEF ≤40% without clinical symptoms of HF.	1
H (HTN)	Resting blood pressure with SBP >140 mmHg and/or DBP >90 mmHg confirmed in at least two separate measurements, or current antihypertensive treatment.	1
A2 (age; additional risk points)	Age ≥75 years.	2
D (diabetes mellitus)	Fasting blood glucose ≥126 mg/dL, or current treatment with antidiabetic medication or insulin.	1
S2 (thromboembolism)	History of ischemic stroke, TIA, peripheral embolism, or pulmonary embolism.	2
V (vascular disease)	Presence of CAD (e.g., prior MI, angina, PCI, or CABG) or PAD (e.g., intermittent claudication, prior surgery or percutaneous intervention on the abdominal aorta or lower extremity arteries, vascular surgery of the thoracic or abdominal aorta, arterial thromboembolism) or complex aortic plaque detected via imaging.*	1
A (age; standard risk points)	Age between 65 years and 74 years.	1

* Complex aortic plaque refers to aortic plaques with mobility, ulceration, pedunculation, or thickness ≥4 mm.

1 CABG, coronary artery bypass grafting; CAD, coronary artery disease; DBP, diastolic blood pressure; HF, heart failure; HTN, hypertension; LVEF, left ventricular ejection fraction; MI, myocardial infarction; PCI, percutaneous coronary intervention; PAD, peripheral arterial disease; SBP, systolic blood pressure; TIA, transient ischemic attack.

Patients scoring 2 or higher are considered to be in high-risk category and should receive OAC therapy, while those scoring 1 are categorized as moderate risk, where therapy should be carefully considered against bleeding risks [29, 30].

In Thai patients, low body weight is an additional risk factor that may warrant consideration, even when their CHA_2_DS_2_-VA score is 0. A sub-analysis from a large registry of AF patients in Thailand found that low body weight is associated with an increased risk of ischemic stroke, independent of the CHA_2_DS_2_-VASc score [35]. However, it is crucial to note that while low body weight may elevate the risk of stroke, it also increases the risk of major bleeding and intracranial hemorrhage. Consequently, the decision to start OAC in this population must be approached with careful consideration.

Given that an individual’s risk of ischemic stroke and thromboembolism changes over time, it is recommended to reassess risk periodically [31].

### Risk of ischemic stroke and thromboembolism in specific patient groups

Patients with AF and coexisting heart conditions, such as hypertrophic cardiomyopathy (HCM), cardiac amyloidosis, moderate-to-severe rheumatic mitral stenosis (MS), or mechanical heart valves [2, 27, 36, 37], are at high risk for ischemic stroke and thromboembolism (Table 7). Thus, OAC is recommended without requiring a CHA_2_DS_2_-VA score assessment.

**Table 7.** Recommendations for assessing the risk of ischemic stroke and thromboembolism in specific patient groups

Recommendation	Class^a^	Evidence^b^
1. It is recommended to use OAC therapy in AF patients with HCM, cardiac amyloidosis, moderate-to-severe rheumatic MS, or mechanical heart valves, regardless of their CHA_2_DS_2_-VA risk score [2, 27, 36, 37].	I	B
2. It is recommended to assess the risk of ischemic stroke and thromboembolism and consider OAC therapy in patients with AFL, similar to those with AF [38].	I	B
3. OAC therapy may be considered in patients with device-detected subclinical AF who are asymptomatic if the duration of subclinical AF is at least 6 min, along with a CHA_2_DS_2_-VA score of ≥4, while also considering individual bleeding risk [39].	IIb	B

1 AF, atrial fibrillation; AFL, atrial flutter; HCM, hypertrophic cardiomyopathy; MS, mitral stenosis; OAC, oral anticoagulant.

a Class of recommendation.

b Level of evidence.

Patients with AFL face an increased risk of ischemic stroke and thromboembolism compared to those without this condition [38]. Therefore, risk assessment and consideration for OAC therapy are also recommended, similar to patients with AF.

### Device-detected subclinical AF

Device-detected subclinical AF refers to AF that occurs in asymptomatic patients not diagnosed through a 12-lead ECG but identified using CIEDs like pacemakers. These patients are at a higher risk for ischemic stroke and thromboembolism compared to those without AF, although their risk is lower than that of patients with clinical AF [40].

The use of OAC therapy in patients with subclinical AF is still under investigation. A sub-analysis of the ARTESIA study [39] indicated that patients with subclinical AF lasting between 6 min and 24 h and a CHA_2_DS_2_-VASc score >4, equivalent to CHA_2_DS_2_-VA >3 [33], had an annual incidence of 2.2% for stroke and thromboembolism. In these cases, apixaban was shown to reduce this risk. However, apixaban did not demonstrate a clear benefit for patients with scores of 4 or lower.

Regular monitoring is essential for all patients to evaluate progression to clinical AF, whether or not they have initiated OAC treatment. Additionally, lifestyle modifications and risk factors management should align with the practices of patients with clinical AF.

## Types of OACs

### DOACs

DOACs inhibit coagulation factors without requiring routine monitoring. In Thailand, approved DOACs include dabigatran (thrombin inhibitor) and apixaban, edoxaban, and rivaroxaban (factor Xa inhibitors). All four DOACs have been shown in randomized clinical trials (RCTs) to be non-inferior to VKAs in preventing ischemic stroke and thromboembolism in patients with AF without moderate-to-severe rheumatic MS or mechanical heart valves [37, 41–45]. Furthermore, DOACs carry a lower risk of intracranial hemorrhage, which is more prevalent in Asian populations [28].

Despite being more expensive, DOACs are more cost-effective due to fewer major bleeding incidents. A study in Thailand found that DOAC-treated patients had lower lifetime healthcare costs and better quality of life than those on VKAs [46]. Therefore, DOACs are recommended as the first-line anticoagulant choice [28] before VKAs in AF patients without moderate-to-severe rheumatic MS and without mechanical heart valves (Table 8). In the COOL-AF registry, DOACs have been shown to achieve better clinical outcomes compared to warfarin [47]. DOACs also had a greater net clinical benefit compared to warfarin [48] and are more cost-effective using the economic evaluation model in Thai population [46].

**Table 8.** Recommendations for the use of OACs in AF patients

Recommendation	Class^a^	Level of evidence^b^
1. It is recommended to use DOACs as the first-line treatment in AF patients without moderate-to-severe rheumatic MS or mechanical heart valves [28], depending on the healthcare setting.	I	A
2. It is recommended to use warfarin as an alternative in AF patients without moderate-to-severe rheumatic MS or mechanical heart valves who are unable to take DOACs [28, 29], depending on the healthcare setting.	I	A
3. It is recommended to use warfarin in AF patients with moderate-to-severe rheumatic MS or mechanical heart valves [37, 45].	I	A
4. It is recommended to maintain an appropriate INR range of 2.0–3.0 in AF patients receiving warfarin [28, 29].	I	B
5. It is advisable to maintain a TTR of at least 65% [49].	IIa	A
6. Lowering the target INR range to 1.5–3.0 may be considered in AF patients aged ≥70 years receiving warfarin to reduce the incidence of major bleeding [50].	IIb	B

1 AF, atrial fibrillation; DOAC, direct oral anticoagulant; INR, international normalized ratio; MS, mitral stenosis; OAC, oral anticoagulant; TTR, time in therapeutic range.

a Class of recommendation.

b Level of evidence.

Each DOAC has distinct pharmacokinetics and dosing regimens (Table 9) [2, 41–44, 51, 52], requiring personalized selection for each patient. While routine drug level monitoring is unnecessary, periodic blood count, liver function, and kidney function assessments should be performed.

**Table 9.** Recommended dosage, pharmacokinetics, and significant drug interactions of OACs [2, 5, 41–44, 51, 52]

Drug	Recommended Dosage	Half-life (h)	Elimination	Significant Drug Interactions
Warfarin	Based on INR (average dose in Thai patients: 2.6 mg/day)	20–60	Renal (0%)	Multiple drugs and food interactions, including: Inhibitors and inducers of CYP2C9, 1A2, or 3A4 Antibiotics Antifungals Herbal supplements
Dabigatran	150 mg BID (reduce to 110 mg BID if age ≥80 years or used with verapamil)	12–17	Renal (80%) Biliary (20%)	- Strong P-gp Inhibitors (e.g., dronedarone, ketoconazole, itraconazole) - Strong P-gp Inducers (e.g., rifampin, carbamazepine, phenytoin)
Rivaroxaban	20 mg QD with food (reduce to 15 mg QD if CrCl is 15–49 mL/min)	5–9 (younger) 11–13 (elderly)	Renal (67%) Liver and biliary system (33%)	- Strong P-gp inhibitors and CYP3A4 Inhibitors (e.g., ketoconazole, itraconazole, ritonavir) - Strong P-gp inducer and CYP3A4 inducer (e.g., rifampin, carbamazepine, phenytoin, phenobarbital)
Apixaban	5 mg BID (reduce to 2.5 mg BID if meeting **2 out of 3** criteria: - Age ≥80 years - Body weight <60 kg - Serum creatinine ≥1.5 mg/dL)	12	Renal (27%–30%) Liver and biliary system (70%)	- Strong P-gp inhibitors and CYP3A4 Inhibitors (e.g., ketoconazole, itraconazole, ritonavir) - Strong P-gp inducer and CYP3A4 inducer (e.g., rifampin, carbamazepine, phenytoin, phenobarbital)
Edoxaban*	60 mg QD (reduce to 30 mg QD if CrCl is 15–50 mL/min, body weight is <60 kg, or used with ciclosporin, dronedarone, erythromycin, or ketoconazole)	10–14	Renal (50%) Liver and biliary system (50%)	- Strong P-gp Inhibitors (e.g., Ritonavir) - Strong P-gp Inducers (e.g., rifampin, carbamazepine, phenytoin, phenobarbital)

* Edoxaban 15 mg QD has been studied in specific subgroups of elderly patients; details can be found in the section on AF management in elderly patients.

1 AF, atrial fibrillation; BID, twice daily; CrCl, creatinine clearance; CYP, cytochrome P450; INR, international normalized ratio; OAC, oral anticoagulant; P-gp, P-glycoprotein; QD, once daily.

In the Asian population, reduced-dose novel oral anticoagulants (NOACs), like Rivaroxaban 15 mg for patients with a creatinine clearance (CrCl) >50 mL/min, are commonly prescribed due to concerns of bleeding. A pharmacokinetic study in Thai patients suggests that this reduced dose may be optimal [53]. However, a meta-analysis of large RCTs [28] indicates that, in the Asian population, while reduced-dose NOACs reduced the risk of major bleeding, they are less effective at preventing ischemic strokes compared to standard doses. Therefore, careful consideration and shared decision-making are essential for choosing the optimal dosing of NOACs to balance bleeding risks with stroke prevention benefits [54].

### VKAs

Warfarin has traditionally been used to lower the risk of thromboembolism in patients with AF, including those with valvular heart disease. Compared to DOACs [51], warfarin has a longer half-life, takes more time to reach therapeutic levels, and requires regular international normalized ratio (INR) monitoring.

The optimal INR range for preventing ischemic stroke and thromboembolism in AF patients is 2.0 to 3.0. However, for those aged ≥70 years, a lower target INR may help reduce the risk of major bleeding, which is more common in Asian populations. The COOL-AF Thailand study, the largest prospective cohort study on AF patients in Thailand, found that the ideal INR range is 2.0–2.99 for patients <70 years and 1.5–2.99 for patients ≥70 years [50].

The efficacy of warfarin relies on proper INR control, typically measured by time in therapeutic range (TTR). The Rosendaal method calculates TTR by assuming a linear relationship between two consecutive INR values [55]. According to data from the COOL-AF Thailand study, a TTR <65% is associated with higher rates of ischemic stroke, thromboembolism, major bleeding, and mortality [49].

Patient responses to warfarin can vary due to genetic differences affecting the *CYP2C9* and *VKORC1* genes. Studies in Thai patients suggest that an initial dose of 2.5–3 mg/day promotes quicker achievement of target INR levels, while 5 mg doses often lead to excessively high INR (Table 9) [52].

In summary, VKA remains the appropriate OAC for AF patients with moderate-to-severe rheumatic MS or mechanical heart valves, while warfarin should only be used when DOACs are not an option (Table 8).

## Risk of bleeding from the use of OACs

### Risk assessment

**Figure 5.**
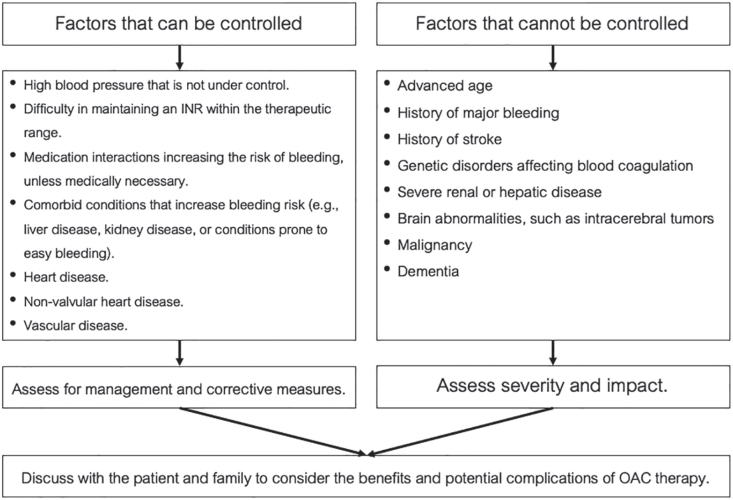
Examples of factors that increase the risk of bleeding from the use of OACs (bleeding risk associated with OACs) and treatment guidelines [2, 56, 57]. INR, international normalized ratio; OAC, oral anticoagulant.

Before starting OAC therapy, it is essential to assess and manage the risk of major bleeding by addressing modifiable factors such as uncontrolled HTN and excessive alcohol consumption (Figure 5), along with any comorbid conditions like liver or kidney disease [2].

While the risk of major bleeding is not a contraindication for anticoagulants, it requires careful consideration and close patient monitoring. For uncontrolled risk factors, evaluate the severity and potential impact of bleeding events and discuss them with the patient and their family to balance benefits and complications.

Regular assessments of bleeding risk should be conducted alongside thromboembolism evaluations during treatment.

### Management of bleeding in patients using OACs

**Figure 6.**
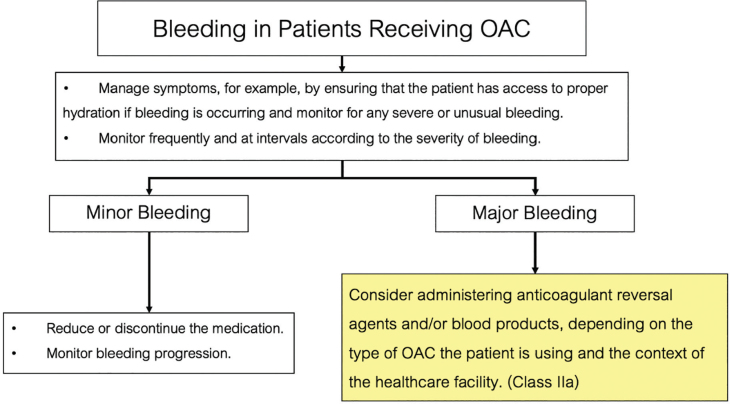
The management guidelines for bleeding in patients receiving OACs. OAC, oral anticoagulant.

For minor bleeding, reducing or temporarily stopping the medication is often sufficient, with close monitoring for any worsening (Figure 6). For major bleeding, immediate care to control the bleeding is crucial, and anticoagulant reversal agents or blood products may be needed. Treatment options depend on the specific OAC and the healthcare facility’s context.

Reversal agents for major bleeding include:

1. Vitamin K reverses the effects of VKA such as warfarin [58].
2. Idarucizumab reverses the effects of direct thrombin inhibitors such as dabigatran [59].
3. Andexanet alfa reverses the effects of factor Xa inhibitors such as apixaban, edoxaban, and rivaroxaban [60].

Blood products for the treatment of major bleeding include:

1. Fresh frozen plasma (FFP) replaces coagulation factors, especially in patients on warfarin [61].
2. Prothrombin complex concentrate (PCC) rapidly increases levels of essential coagulation factors (e.g., factor II, VII, IX, and X). PCC is frequently used in severe bleeding cases associated with warfarin or DOAC therapy [61, 62].

Managing major bleeding in patients on OACs requires a multidisciplinary team, including hematology and cardiology specialists and emergency physicians, to ensure a comprehensive assessment and appropriate decision-making. The choice of reversal agents or blood products depends on the context of each healthcare facility (Table 10).

**Table 10.** Recommendations for risk assessment and management of bleeding

Recommendation	Class^a^	Evidence^b^
1. It is recommended to assess the risk of major bleeding before starting OACs and reassess periodically [2].	I	B
2. It is recommended to stop OACs when major bleeding occurs and cannot be controlled, until the underlying cause is identified and controlled.	I	C
3. Consider using reversal agents for major bleeding, such as vitamin K for warfarin, idarucizumab for thrombin inhibitors, or andexanet alfa for factor Xa inhibitors, depending on the healthcare setting [58–60].	IIa	B
4. Consider using blood products, such as FFP or PCC, for managing major bleeding, depending on the healthcare setting [61, 62].	IIa	C
5. Consider percutaneous LAAO for AF patients who have an absolute contraindication to OACs [63].	IIb	C

1 AF, Atrial fibrillation; FFP, fresh frozen plasma; OAC, oral anticoagulant; PCC, prothrombin complex concentrate; LAAO, left atrial appendage occlusion.

a Class of recommendation.

b Level of evidence.

### Resumption of OAC after bleeding

The decision to resume OAC and the appropriate timing for restarting the medication depend on several factors, such as the severity of the bleeding, the cause of the bleeding, and the risk of ischemic stroke and thromboembolism. These factors need to be discussed carefully and in detail with the patient and their family, considering the patient’s needs and expectations.

Data from studies related to resuming OACs are limited, with most being retrospective studies. The key considerations are as follows:

1. Patients with gastrointestinal (GI) bleeding: Studies have found that resuming OACs, even though associated with a higher risk of recurrent bleeding, is also linked to a reduced risk of ischemic stroke and thromboembolism, as well as a decreased mortality rate. The study suggests that the appropriate time to restart OACs is 2 weeks after the bleeding event [64].
2. Patients with cerebral bleeding: Studies indicate that resuming OACs within 3 months is associated with a lower risk of ischemic stroke and thromboembolism compared to those who do not resume OAC, and it does not increase the risk of recurrent cerebral bleeding [65].
3. Patients with mechanical heart valves and cerebral bleeding: It has been found that the appropriate time to resume OACs is approximately 1–2 weeks after the bleeding event.
4. Patients with primary brain tumors: The use of OACs increases the risk of cerebral bleeding by 2.6 times compared to patients not using OAC. However, in patients with metastatic brain tumors, the use of OACs does not increase the risk of cerebral bleeding [66].

The decision to resume OACs should consider the severity, cause, and treatment approach for the bleeding event. It depends on a shared decision-making process between the attending physician and the patient. However, for causes of major or life-threatening bleeding that cannot be treated or corrected, the risk of continued bleeding may outweigh the benefits of preventing thromboembolism.

### Left atrial appendage occlusion

Left atrial appendage occlusion (LAAO) aims to prevent blood clots that form in the left atrial appendage (LAA) from traveling into the bloodstream, causing ischemic stroke and thromboembolism. LAAO can be performed through surgery (surgical LAAO) or a device inserted through the venous system (percutaneous LAAO).

Research on LAAO is ongoing. Current data suggest that percutaneous LAAO may be suitable for patients who cannot use OACs, especially in cases of uncorrectable bleeding [67]. Important factors include the device type, procedural complications, long-term data limitations, medical expertise, and hospital resources. These factors should be discussed with the patient and their family to address individual needs and expectations.

### Heart rate control with medications

AF can cause various responses in the ventricular heart rate or pulse. These include a slow heart rate (<50 bpm), a normal heart rate (between 50 bpm and 100 bpm, typically averaging 60–80 bpm), and a fast heart rate (>100 bpm). Patients with a fast heart rate are often those with more significant symptoms, such as palpitations, chest pain, and sometimes shortness of breath. Importantly, a fast heart rate can lead to HF due to prolonged periods of rapid ventricular rhythm, which is known as tachycardia-induced cardiomyopathy. Even if the pulse is not extremely fast, an irregular ventricular rhythm can still lead to HF.

Therefore, heart rate control with medication is an essential foundational treatment to reduce symptoms from a rapid heart rate and prevent long-term complications [68]. However, we still lack sufficient data to determine the optimal target heart rate for treatment and the best method for achieving it [69]. The following findings from important studies may guide clinicians in appropriately treating their patients:

1. **RACE II Study**: An RCT comparing lenient rate control (resting heart rate <110 bpm) with strict rate control (resting heart rate <80 bpm) in patients with permanent AF. The study found no significant difference in cardiovascular death, hospitalization due to HF, stroke, systemic embolism, or life-threatening arrhythmic events between the two groups, suggesting that strict rate control is not a superior approach. However, the study had limitations, as the heart rate differences between the two groups were about 10 bpm [70].
2. **HOT CAFE Study**: An RCT comparing rate control using medication with rhythm control, which involved electrical cardioversion and antiarrhythmic drugs, in patients with persistent AF. The study found that all-cause mortality, the number of thromboembolic events, and major bleeding were similar between the rate control and rhythm control groups [71].
3. **Meta-Analysis**: A meta-analysis of RCTs and observational studies comparing rate control with rhythm control found no significant difference in overall mortality and mortality due to heart disease between the two treatment groups [72].
4. **ORBIT-AF Study**: A prospective cohort study in patients with heart rates above or below 65 bpm. The study showed a J-shaped relationship, with mortality rates increasing for both higher and lower heart rates compared to the 65 bpm group. Patients with either a heart rate higher or lower than 65 bpm had higher overall mortality rates [73] and a higher incidence of HF [73]. However, the study did not provide a definitive heart rate that would best reduce mortality, but it suggested that both excessively high and low heart rates resulted in poorer treatment outcomes.
5. **Study on Heart Rate and Outcomes in Patients with Sinus Rhythm and AF with Heart Failure with Reduced Ejection Fraction (HFrEF) [74]:** The study found that for patients with AF and HFrEF, a resting heart rate >86 bpm did not lead to higher mortality compared to those with a resting heart rate <72 bpm. However, in patients with sinus rhythm and HFrEF, an increase in heart rate by 10 bpm was associated with a 31% higher mortality rate from heart disease and HF. This study suggests that for patients with sinus rhythm and HFrEF, strict heart rate control, such as maintaining a heart rate <70 bpm, is recommended. In contrast, for AF patients with HFrEF, there is no clear evidence that reducing heart rate will decrease mortality rates.

## Objectives of rate control therapy

1. Improve left ventricular ejection fraction (LVEF) by treating tachycardia-induced cardiomyopathy.
2. Reduce the risk of inappropriate shocks in patients with an ICD.
3. Alleviate symptoms associated with a rapid heart rate.
4. Reduce hospitalization rates in patients with tachycardia-bradycardia syndrome.
5. Enhance response to cardiac resynchronization therapy (CRT).

**Figure 7.**
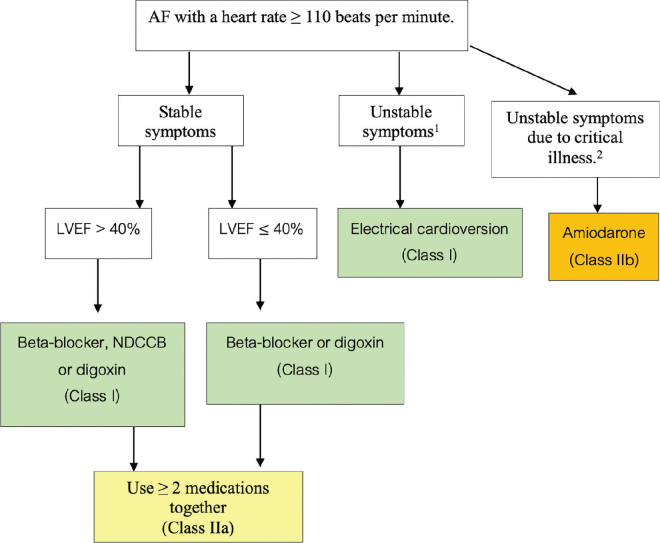
Management guideline for AF patients using rate control. ^1^Unstable symptoms refer to conditions, such as shock, acute HF, severe chest pain associated with ACS, or respiratory failure. ^2^Critical illness refers to conditions, such as sepsis, septic shock, or respiratory failure from lung disease. ACS, acute coronary syndromes; AF, atrial fibrillation; HF, heart failure; LVEF, left ventricular ejection fraction; NDCCB, non-dihydropyridine calcium-channel blocker.

Recommendations and guidelines for rate control treatment in patients with AF using medications are shown in Table 11 and Figure 7, while the medications used for rate control therapy are shown in Table 12.

**Table 11.** Recommendations for rate control therapy with medication in patients with AF [1, 2, 70, 75–79]

Recommendation	Class^a^	Evidence^b^
1. It is recommended to use medication for rate control in patients with acute symptoms, along with rhythm control therapy, to reduce symptoms associated with a rapid heart rate.	I	A
2. It is recommended to select medication for rate control based on the patient’s comorbidities and LVEF.	I	A
3. Beta-blockers, NDCCBs (verapamil or diltiazem), or digoxin should be used for rate control in patients with AF and LVEF >40%.	I	A
4. Beta-blockers or digoxin should be used for rate control in patients with AF and LVEF ≤40%.	I	A
5. It may be considered to use a combination of two or more medications for rate control when the heart rate cannot be controlled <110 bpm, with caution for bradycardia and regular monitoring of heart rate.	IIa	C
6. It may be considered to use rate control medication to achieve a target heart rate of <110 bpm or lower if symptoms persist, aiming to reduce symptoms from rapid heart rates or eliminate symptoms while avoiding bradycardia.	IIa	B
7. It may be considered to use digoxin in combination with other rate control medications or as a standalone therapy.	IIa	B
8. It may be considered to monitor serum digoxin levels, maintaining a blood level of ≤1.2 ng/mL.	IIa	B
9. It may be considered to perform AVN ablation combined with pacemaker implantation in patients who do not respond to medication or standard therapies and cannot undergo intensive rate and rhythm control or have limitations for such treatments.	IIa	B
10. It may be considered to perform AVN ablation combined with CRT or CSP in patients with permanent AF who have significant symptoms and a history of HF hospitalizations, to reduce symptoms, hospital readmissions due to HF, and mortality.	IIa	B
11. Rate control therapy may be considered in patients with valvular heart disease in the same manner as in patients without valvular heart disease.	IIa	C
12. Amiodarone may be considered for rate control in patients with AF and HFrEF who have limitations for beta-blocker and digoxin use.	IIb	C
13. Amiodarone, esmolol, or intravenous digoxin may be considered for rate control in patients with hemodynamic instability (e.g., hypotension or significantly reduced LVEF).	IIb	C

1 AF, atrial fibrillation; AVN, atrioventricular node; CRT, cardiac resynchronization therapy; CSP, conduction system pacing; HF, heart failure; HFrEF, heart failure with reduced ejection fraction; LVEF, left ventricular ejection fraction; NDCCB, non-dihydropyridine calcium-channel blocker.

a Class of recommendation.

b Level of evidence.

**Table 12.** Medications used for rate control therapy

Medication type	IV administration*	Oral Dose*	Precautions
Beta-blockers			1. In patients with asthma, avoid non-selective beta-blockers. 2. Avoid in acute HF.
Esmolol	500 μg/kg IV bolus within 1 min, followed by 50–300 μg/kg/min	N/A	Used for urgent rate control.
Metoprolol XL (succinate)	N/A	50–200 mg once daily	
Bisoprolol	N/A	1.25–20 mg once daily	
Nebivolol	N/A	2.5–10 mg once daily	
Carvedilol	N/A	3.125–50 mg twice daily	
Metoprolol tartrate	N/A	25–100 mg twice daily	Not recommended in LVEF ≤40%.
Atenolol	N/A	25–100 mg once daily	Not recommended in LVEF ≤40%.
NDCCB			Not recommended in patients with LVEF ≤40%
Verapamil	2.5–10 mg IV bolus within 5 min	40 mg twice daily up to 480 mg (extended release) once daily	
Diltiazem	0.25 mg/kg IV bolus over at least 5 min, followed by 5–15 mg/h	60 mg three times daily up to 360 mg (extended release) once daily	
Other medications			
Digoxin	0.5 mg IV bolus (0.75–1.5 mg >24 h, divided into 2–3 doses)	0.0625–0.125 mg once daily	Consider checking serum drug levels and use with caution in patients with CKD. Dose adjustment is needed.
Amiodarone	300 mg IV in 250 mL 5% dextrose >30–60 min, then 900–1,200 mg IV in 24 h in 500–1,000 mL 5% dextrose. A central line may be considered for higher concentrations.	200 mg once daily after IV loading, or loading dose of 200 mg three times a day for 4 weeks, then 200 mg (or less) once daily	For rhythm control dosing, refer to the rhythm control section.
Magnesium	3–5 g IV in 10–20 min		1. Not recommended for POAF. 2. Common side effects include flushing. 3. Rare side effects include hypotension; monitoring is needed after administration.

* The dose can be adjusted as appropriate.

1 CKD, chronic kidney disease; HF, heart failure; i.v., intravenous; LVEF, left ventricular ejection fraction; min, minutes; N/A, not available or not widely available; NDCCB, non-dihydropyridine calcium-channel blocker; POAF, post-operative atrial fibrillation.

### Digoxin

There has been increasing discussion about the role of digoxin for rate control. In a systematic review and meta-analysis of RCTs and observational studies comparing digoxin use with no medication, the overall mortality rate was not significantly different between those who received the medication and those who did not in the RCTs. However, the hospitalization rate was lower in all types of studies for patients who received digoxin. The populations studied included both patients with HF and those without this condition [80]. Although other observational studies found an increased mortality rate [81], the RATE-AF study, the most recent research comparing digoxin with bisoprolol in patients with permanent AF, found no difference in overall quality of life after 6 months between those receiving digoxin and those receiving bisoprolol. However, patients receiving digoxin experienced fewer side effects, fewer symptoms, and a greater reduction in B-type natriuretic peptide (BNP) [82].

### Magnesium

The effect of magnesium (Mg) on rate control therapy is due to its ability to inhibit calcium channels, which affects cell entry at the atrioventricular node (AVN), resulting in a reduced conduction speed at this level. Studies that have compiled data on the use of intravenous Mg have shown that it enhances the effectiveness of rate control and increases the likelihood of restoring sinus rhythm. The results are more pronounced when magnesium levels are lower than normal. However, the definitions used in studies evaluating the effect of Mg on rate control therapy vary significantly, ranging from <90 bpm to <110 bpm or a reduction in heart rate by >20% [83, 84]. Current treatment guidelines recommend a target of <110 bpm for rate control. Furthermore, the dosages used in these studies ranged from 2.5 g to 10 g. A subgroup analysis comparing high-dose (>5 g) and low-dose (≤5 g) magnesium found that the group receiving ≤5 g of magnesium had a higher chance of restoring sinus rhythm [85]. Since most studies have used 5 g of Mg [83, 84], it is recommended to use 5 g of magnesium for rate control, and it can be used even in patients with normal magnesium levels.

### AVN ablation with pacemaker, CRT, or conduction system pacing

In certain patients with paroxysmal AF who are difficult to manage with rate and rhythm control under normal conditions, either with medication or catheter ablation, and still experience significant symptoms from AF, AVN ablation can be performed to induce iatrogenic complete heart block, preventing electrical conduction from the atrium to the ventricle. A pacemaker is then implanted in cases where the LVEF is normal to prevent bradycardia [86]. This approach also results in a stable heart rate and fewer complications, with a low incidence of reduced LVEF, and may even improve LVEF. However, this treatment is most appropriate for elderly patients, as they will depend on the pacemaker. AVN ablation is not recommended for younger patients.

Additionally, AVN ablation with CRT or conduction system pacing (CSP) is performed in cases where LVEF <50%, aiming to reduce heart rate and achieve regularity, which may also slow the decline in LVEF and improve HF. Key studies supporting this treatment include the APAF-CRT trial [87, 88], which compared AVN ablation with CRT implantation in patients without a broad QRS complex (QRS >120 ms), unlike the standard indication for CRT. This study showed a significant reduction in mortality and HF-related hospitalizations in patients receiving AVN ablation and CRT compared to those on rate control alone.

Recent data also support AVN ablation combined with CSP as an alternative treatment, potentially reducing HF, although safety concerns related to lead stability still need confirmation through further RCTs.

## Summary

Rate control therapy is an essential treatment that should be considered alongside rhythm control. The control strategy depends on whether the goal is acute (urgent) or long-term control. There are three main drug classes for rate control: beta-blockers, non-dihydropyridine calcium-channel blockers (NDCCBs) such as verapamil or diltiazem, and digoxin, which can be used in both acute and long-term settings. Mg can also be used as an additional option for acute rate control. The choice of medication should primarily be based on LVEF. The efficacy of the three main drug classes is comparable, and treatment should be initiated one drug at a time, with heart rate and blood pressure monitoring, aiming to maintain a heart rate of no less than 50–60 bpm. If adequate heart rate control and symptom management are not achieved with a single agent, a combination of two drugs may be considered with close monitoring to prevent bradycardia and its associated symptoms. In certain cases, three-drug combinations may be used for heart rate control, usually in patients with a pacemaker, to prevent excessive bradycardia. Additional considerations include dose adjustment when using verapamil with dabigatran, requiring a reduction to 110 mg twice daily, and the use of amiodarone for rate control based on appropriate indications. If there are no contraindications, OAC should be considered, as amiodarone can also be used for rhythm control in pharmacological cardioversion, which may increase the risk of ischemic stroke and systemic thromboembolism.

### Management to maintain sinus rhythm with medications and cardioversion

The treatment for symptom reduction in AF through rhythm control consists of two stages:

**Stage 1:** The initial treatment to reduce symptoms from AF is rate control, which involves the use of atrioventricular (AV) nodal blocking drugs. The goal of rate control treatment is to maintain the heart rate <110 bpm, or even lower, depending on the patient’s symptoms.
**Stage 2:** After rate control, the next step is to determine whether the patient requires rhythm control. The two main treatment options are:
  (1) The long-term use of antiarrhythmic drugs and
  (2) Non-pharmacological methods, such as catheter ablation.

These approaches aim to restore and maintain sinus rhythm to improve symptoms and prevent further complications.

### Patient group considerations for rhythm control treatment

**Figure 8.**
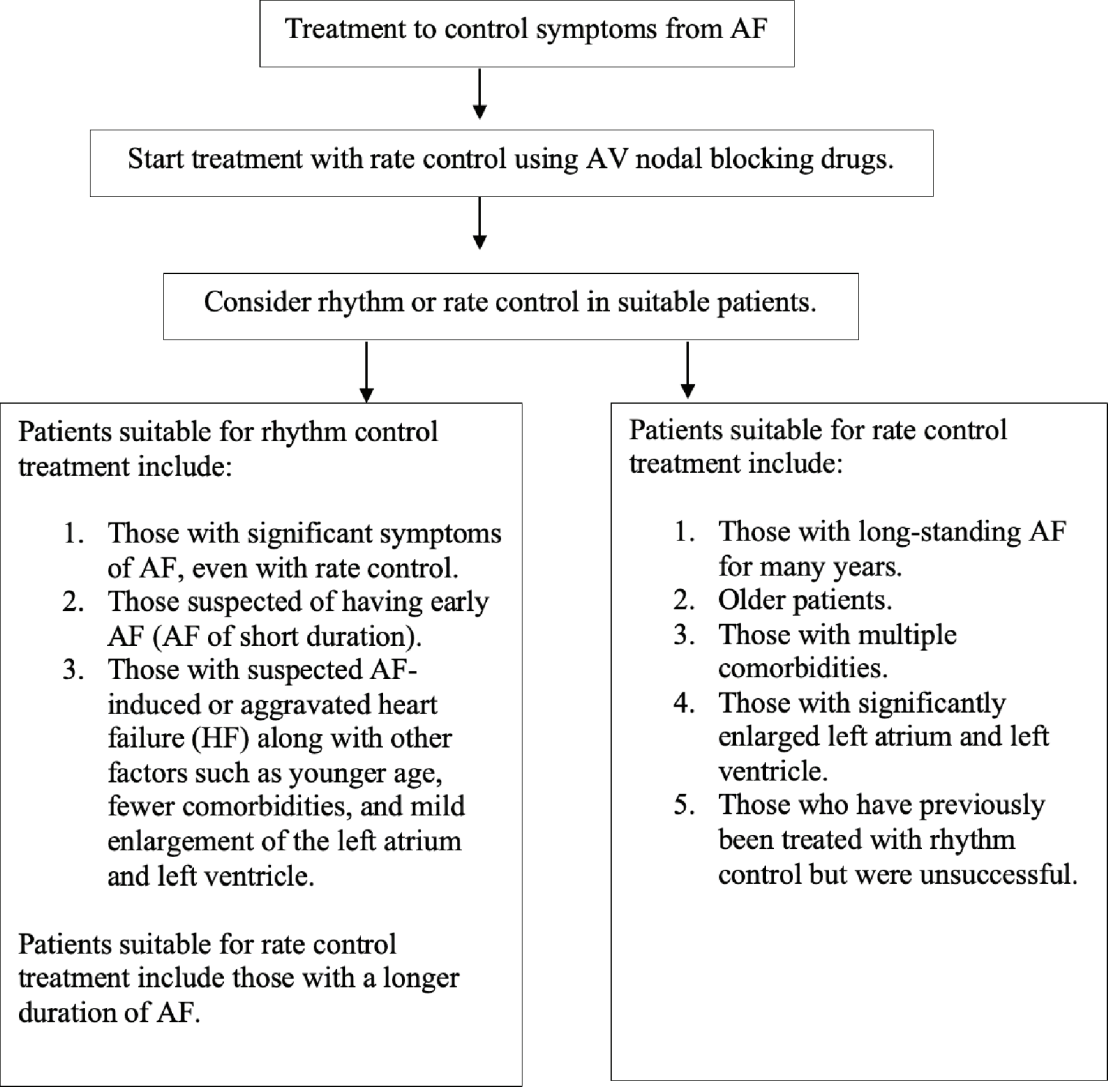
Approach for choosing the method of treatment to control symptoms from AF. AF, atrial fibrillation; AV, atrioventricular; HF, heart failure.

1. **Symptomatic AF:** Patients who continue to experience significant symptoms from AF, even after appropriate rate control.
2. **Early AF:** Patients who have been diagnosed with AF for a relatively short period (<1 year).
  The EAST-AFNET 4 study [89] found that for patients with AF of short duration (<1 year), rhythm control treatment significantly reduced the incidence of overall cardiovascular death, stroke, hospitalization due to HF, and acute coronary syndromes (ACS) compared to rate control treatment (Table 13 and Figure 8). However, the AFFIRM study [90], published in 2002, focused on patients with long-standing AF and found no benefit of rhythm control with antiarrhythmic drugs compared to rate control. Therefore, it is recommended that patients who have been diagnosed with AF recently (early AF) should be considered for rhythm control as the first choice, as they are likely to benefit more than patients with long-standing AF.
3. **Patients with HFrEF:** Specifically, those with tachycardia-induced or AF-induced cardiomyopathy, as this is a reversible cause of HF. Furthermore, in patients with AF and HFrEF, if AF is thought to worsen HF symptoms (AF-aggravated HF), studies such as CASTLE-AF [91] and CASTLE-HTx [92] showed that catheter ablation to treat AF can significantly reduce mortality and hospitalization due to HF compared to medication. Therefore, AF catheter ablation is recommended for this group after assessing the risks and benefits of the procedure with the patient and their family.

**Table 13.** Recommendations for selecting patients for rhythm control treatment

Recommendation	Class^a^	Evidence^b^
1. Rhythm control should be considered for patients who still have significant symptoms of AF, even after appropriate rate control (symptomatic AF).	IIa	B
2. Rhythm control should be considered for patients who have had AF for a short duration (early AF).	IIa	B
3. Rhythm control is recommended for patients with HFrEF caused by tachycardia-induced cardiomyopathy, with catheter ablation being considered after evaluating the benefits and risks of the procedure with the patient.	I	B
4. Rhythm control should be considered for patients with pre-existing HFrEF who have worsening symptoms due to AF, with catheter ablation being considered after evaluating the benefits and risks of the procedure with the patient.	IIa	B

1 AF, atrial fibrillation; HFrEF, heart failure with reduced ejection fraction.

a Class of recommendation.

b Level of evidence.

However, the decision to choose rhythm control should also take into account other factors, such as younger age, fewer comorbidities, and less enlargement of the left atrium (LA) and left ventricle. These factors suggest a higher likelihood of success in restoring sinus rhythm. It is important to consult with the patient and their family about the benefits and risks of each treatment approach, ensuring shared decision-making in the treatment selection process [2].

### Rhythm control treatment consists of two steps

**Figure 9.**
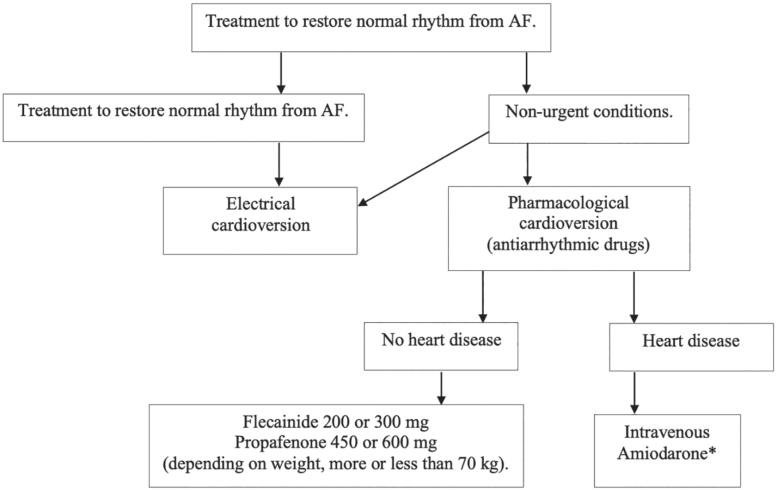
Guidelines for cardioversion in patients with AF. *The intravenous dose of amiodarone is 300 mg administered intravenously >30–60 min, followed by 900–1,200 mg administered within 24 h. AF, atrial fibrillation.

**Step 1:** Cardioversion to restore AF to sinus rhythm. This includes patients who have been newly diagnosed with AF and where the physician intends to restore sinus rhythm (acute rhythm control), or persistent AF patients who have been assessed as suitable candidates for longterm rhythm control treatment [93]. For paroxysmal AF patients who spontaneously revert to sinus rhythm, cardioversion is not necessary. The approach to cardioversion in AF patients is shown in Figure 9.

**Figure 10.**
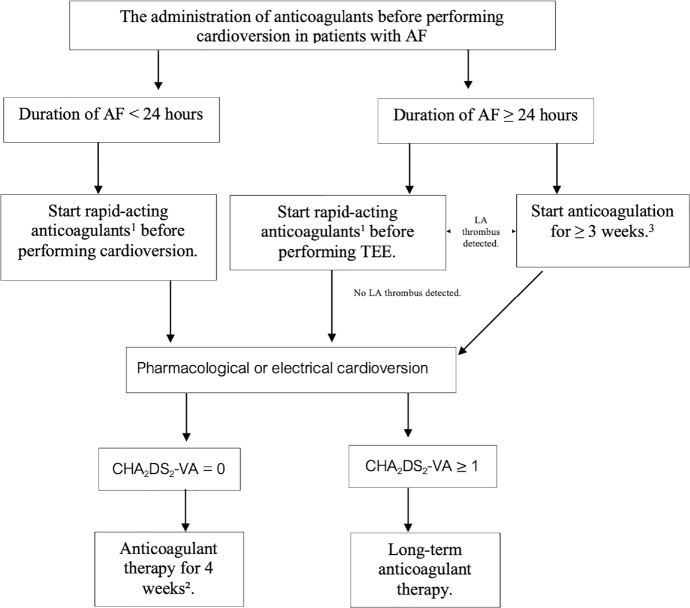
Anticoagulants before performing cardioversion in patients with AF. 1Fast-acting anticoagulants include UFH, LMWH, and DOAC. ^2^In cases where the duration of AF is <24 h and the risk of thromboembolism is low, there may be an option not to administer anticoagulants after cardioversion. ^3^In cases using warfarin, an INR level ≥2.0 should be maintained for ≥3 weeks. For DOAC, the medication should be taken continuously for 3 weeks. AF, atrial fibrillation; DOAC, direct oral anticoagulant; LA, left atrium; LMWH, low molecular weight heparin; TEE, transesophageal echocardiography; UFH, unfractionated heparin.

Cardioversion may increase the risk of thromboembolism, with the risk being higher depending on the duration of AF and individual thromboembolic risk, which is assessed by the CHA_2_DS_2_-VA score. The recommendation for OAC before attempting cardioversion is shown in Figure 10 [94, 95].

**Step 2 :** Maintenance of sinus rhythm, which involves long-term antiarrhythmic drug therapy or catheter ablation.

### Selection of antiarrhythmic drugs for long-term rhythm control

**Figure 11.**
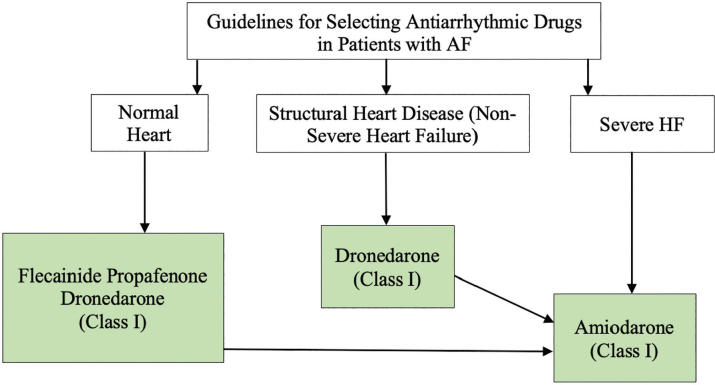
Selection of long-term antiarrhythmic drugs in patients with AF. AF, atrial fibrillation; HF, heart failure‥

In Thailand, there are four oral antiarrhythmic drugs available for the treatment of AF: flecainide and propafenone, which belong to the Class IC antiarrhythmic drugs, and dronedarone and amiodarone, which are classified as Class III antiarrhythmic drugs (Table 14 and Figure 11). The selection of these drugs prioritizes safety over treatment efficacy. Among them, amiodarone has the highest efficacy in preventing AF recurrence but also carries the highest risk of long-term side effects compared to other drugs [96]. Therefore, amiodarone is not recommended as the first-line choice for AF patients unless other antiarrhythmic drugs are contraindicated or cause intolerable side effects. The recommendations for the use of antiarrhythmic drugs in different AF patient groups are as follows:

1. Patients without structural heart disease—Flecainide, propafenone, or dronedarone are recommended as first-line choices due to their relatively lower long-term side effects [2]. If these drugs are ineffective or contraindicated, amiodarone can be considered, or catheter ablation can be selected as an alternative treatment.
2. Patients with structural heart disease—This includes those with coronary artery disease (CAD) or left ventricular hypertrophy due to HTN but without severe HF. Dronedarone is recommended as the first-line drug [2, 97]. If ineffective or contraindicated, amiodarone can be used, or catheter ablation can be considered.
3. Patients with severe HF—This includes patients with HFrEF classified as New York Heart Association (NYHA) functional class III or IV or those who have experienced decompensated HF within the past month. Amiodarone is the recommended drug, as it is the only antiarrhythmic agent proven to be safe in this patient group [98]. Alternatively, catheter ablation may be considered.

**Table 14.** Recommendations for selecting antiarrhythmic drugs for long-term rhythm control

Recommendation	Class^a^	Evidence^b^
1. It is recommended to use flecainide, propafenone, or dronedarone as the first-line choice in patients without I structural heart disease.	I	A
2. It is recommended to use dronedarone in patients with other heart diseases, such as CAD or left ventricular hypertrophy due to HTN, but without severe HF.*	I	A
3. It is recommended to use amiodarone in patients with severe HF* or in cases where other antiarrhythmic drugs I cannot be used.	I	A
4. It is advisable to consider AV nodal blocking drugs, such as beta-blockers, verapamil, or diltiazem, in conjunction with flecainide or propafenone to prevent AFL with 1:1 AV conduction.	IIa	C

* Severe HF refers to patients with HFrEF who have a NYHA functional class III or IV or have experienced decompensated HF within the past month.

1 AFL, atrial flutter; AV, atrioventricular; CAD, coronary artery disease; HF, heart failure; HFrEF, heart failure with reduced ejection fraction; HTN, hypertension; NYHA, New York Heart Association.

a Class of recommendation.

b Level of evidence.

The dosage and potential side effects of antiarrhythmic drugs are presented in Table 15.

**Table 15.** Dosage of antiarrhythmic drugs and side effects

Drug	Initial dose^a^	Long-term dose^a^	Monitoring	Side effects
Flecainide	50 mg/dose, taken twice daily	100–150 mg/dose, taken twice daily	QRS duration^b^ at 2–4 weeks, and when adjusting the dosage	AFL with 1:1 AV conduction, therefore, AV nodal blocking drugs (e.g., beta-blockers, verapamil, diltiazem) must always be coadministered
Propafenone	150 mg/dose, taken three times daily	300 mg/dose, taken three times daily	QRS duration^b^ at 2–4 weeks, and when adjusting the dosage	AFL with 1:1 AV conduction; therefore, AV nodal blocking drugs (e.g., beta-blockers, verapamil, diltiazem) must always be coadministered
Dronedarone	400 mg/dose, taken twice daily	400 mg/dose, taken twice daily	QTc interval^c^, Liver function test periodically (e.g., every 6 months as appropriate)	- Torsade de Pointes (rare) - Bradycardia - Liver toxicity (rare)
Amiodarone	Loading Dose: 6–10 g^d^ Inpatients: 300 mg IV >30–60 min, followed by 900–1,200 mg IV >24 h, then oral 200 mg/dose, three times daily until total dose reaches 6–10 g	Maintenance Dose: 100–200 mg/dose, taken once daily	QTc interval^c^, Liver function test, Thyroid function test periodically (e.g., every 6 months as appropriate), Chest X-ray if respiratory symptoms occur	- Torsade de Pointes (rare) - Bradycardia - Blue-gray discoloration - Corneal microdeposit - Liver toxicity - Thyroid dysfunction - Pulmonary fibrosis
	Outpatients: 200 mg/dose, three times daily until total dose reaches 6-10 g			

a The dosage may be adjusted as appropriate.

b If QRS duration increases by >25% from baseline, consider reducing the dose of flecainide or propafenone or discontinuing the drug.

c If QTc interval exceeds 500 ms, consider reducing the dose of dronedarone or amiodarone or discontinuing the drug.

d Amiodarone has an average half-life of 58 days, necessitating an initial high-dose (loading dose) followed by a lower long-term maintenance dose.

1 AFL, atrial flutter; AV, atrioventricular; QTc, corrected QT.

### Catheter ablation for AF treatment

Catheter ablation is a procedure used to prevent recurrence, reduce AF burden, and improve quality of life in patients with paroxysmal, persistent, and permanent AF who continue to experience symptoms despite receiving rhythm control medications or who suffer from adverse effects of antiarrhythmic drugs. Numerous studies indicate that early catheter ablation is effective for paroxysmal AF, with similar risks to antiarrhythmic drugs. However, for persistent AF, there is currently no conclusive evidence that catheter ablation is superior to antiarrhythmic drug therapy. Additionally, catheter ablation may play a role in patients experiencing bradycardia after AF converts to sinus rhythm (post-conversion pause), helping reduce symptoms and the need for a pacemaker [99] (Table 16).

**Table 16.** Recommendations for catheter ablation in AF patients

Recommendation	Class^a^	Evidence^b^
1. Patients should be involved in decision-making regarding catheter ablation after receiving counseling on the risks and benefits of the procedure, as well as the risk of recurrence.	I	C
2. Catheter ablation is recommended for patients with symptomatic paroxysmal or persistent AF who do not respond to antiarrhythmic drugs or cannot tolerate them, to reduce symptoms, recurrence, and disease progression.	I	A
3. Catheter ablation may be considered as a first-line treatment, depending on the institutional context, after shared decision-making with the patient, for symptomatic paroxysmal AF to reduce symptoms, recurrence, and disease progression.	IIa	A
4. Catheter ablation may be considered as a first-line treatment after shared decision-making with the patient, for symptomatic persistent AF to reduce recurrence and disease progression.	IIb	C
5. Catheter ablation is recommended for patients with AF and HFrEF who are highly likely to have tachycardia-induced cardiomyopathy to improve LVEF.	I	B
6. Catheter ablation should be considered for selected patients with AF and HFrEF to reduce hospitalizations due to pulmonary congestion and improve survival.	IIa	B
7. Catheter ablation should be considered for AF patients experiencing bradycardia after AF returns to sinus rhythm, to improve symptoms and avoid pacemaker implantation.	IIa	C
8. Repeat catheter ablation should be considered for AF patients who experience recurrence after an initial procedure, if they had symptomatic improvement after the first ablation, or if the first attempt was unsuccessful, to reduce symptoms, recurrence, and disease progression.	IIa	B

1 AF, atrial fibrillation; HFrEF, heart failure with reduced ejection fraction; LVEF, left ventricular ejection fraction.

a Class of recommendation.

b Level of evidence.

Pulmonary vein isolation (PVI) is the primary approach for catheter ablation [100]. Currently, there are newer ablation techniques, including cryoballoon ablation and pulsed field ablation, which have shown effectiveness comparable to radiofrequency ablation. These three methods demonstrate similar success rates and risks without significant statistical differences.

Cryoballoon ablation is a highly effective and safe procedure. Its advantages include a shorter procedure time (reduced from 3–4 h to 2–3 h), lower risk of injury to surrounding tissues, reduced pain, and fewer complications. However, a limitation is that this method is only used for PVI, so if abnormalities exist outside this region, it may not be applicable. There is also a 2% risk of phrenic nerve injury, which typically resolves within 3–6 months. Given its effectiveness and safety, cryoballoon ablation is a good treatment option for AF. However, patients should consult with specialists to discuss benefits, limitations, and suitability for treatment.

Pulsed field ablation is a new technique that uses high-intensity electrical energy to selectively destroy abnormal myocardial cells without relying on heat or cold, as traditional methods do. This method provides high precision, allowing targeted ablation while sparing surrounding tissues, such as the esophagus, nerves, and coronary arteries, thereby reducing complications. This makes it superior to other ablation methods. Additionally, it shortens the procedure time due to its efficiency in rapidly ablating abnormal tissues. However, since this is a new technique, further research is required to confirm its long-term safety and efficacy. Patients should consult a specialist to assess suitability and stay updated on emerging clinical data.

The selection of rhythm control therapy should be based on patient characteristics. In asymptomatic AF patients, catheter ablation should be cautiously considered after shared decision-making with the patient. In HFrEF patients, catheter ablation can reduce recurrence, improve cardiac function, and lower mortality rates [101, 102]. However, if the patient has an enlarged LA or significant fibrosis, the benefit of catheter ablation may be limited [103]. For patients with heart failure with preserved ejection fraction (HFpEF), catheter ablation is not as beneficial as in those with HFrEF [104].

The complication rate of catheter ablation is approximately 2.9%–7.2%, with a mortality rate <0.1% [105]. Currently, AF monitoring after catheter ablation has advanced with the use of smartwatches [106] and ILRs to track AF burden and frequency before and after ablation.

Antiarrhythmic drug therapy for 2–3 months after catheter ablation can help reduce early AF recurrence [67], but it does not impact long-term recurrence rates [107]. Repeat catheter ablation may be considered for patients who continue to experience significant symptoms following the first procedure [108].

### Use of OACs in patients undergoing catheter ablation

The presence of a thrombus in the LA is a contraindication for catheter ablation, as it significantly increases the risk of ischemic stroke (Table 17). Therefore, patients at risk of ischemic stroke who are scheduled for catheter ablation should receive OACs for at least 3 weeks before the procedure [109].

**Table 17.** Recommendations for the use of OACs in patients with AF undergoing catheter ablation

Recommendation	Class^a^	Evidence^b^
1. It is recommended to initiate OAC for at least 3 weeks before catheter ablation in AF patients at risk of thromboembolism to prevent ischemic stroke.	I	C
2. Patients undergoing catheter ablation should continue receiving OACs to prevent ischemic stroke during the procedure.	I	A
3. OAC is recommended for at least 2 months after catheter ablation in all patients, regardless of post-procedure heart I rhythm or thromboembolism risk, to prevent ischemic stroke following the procedure.	I	C
4. Long-term continuation of OAC is recommended after catheter ablation in patients at risk of thromboembolism, regardless of the success of rhythm control, to prevent ischemic stroke.	I	C
5. A TEE should be considered to assess for intracardiac thrombus before catheter ablation in patients at high thromboembolism risk, even if they are already on OAC.	IIa	B

1 AF, atrial fibrillation; OAC, oral anticoagulant; TEE, transesophageal echocardiography.

a Class of recommendation.

b Level of evidence.

Thrombus detection in the heart can be performed using various imaging modalities such as transesophageal echocardiography (TEE), intracardiac echocardiography, or computed tomography (CT). The prevalence of cardiac thrombi is higher in patients at increased risk of ischemic stroke and is more commonly found in persistent AF than in paroxysmal AF [110]. Additionally, certain patient populations, such as those with HCM, amyloidosis, or rheumatic heart disease (RHD), have a higher risk of thrombus formation.

Before catheter ablation, discontinuing OACs should generally be avoided. In patients taking once-daily OACs, switching the dose to the evening on the day of catheter ablation may be considered. Studies comparing discontinuation of OACs on the morning of catheter ablation with continued administration found no significant differences in efficacy and safety [111].

During catheter ablation, intravenous anticoagulation is administered [112]. After the procedure, once hemostasis is achieved, OAC should be continued for at least 2 months following catheter ablation [109]. Beyond these 2 months, the decision to continue OAC depends on the patient’s risk of thromboembolism, assessed using the CHA_2_DS_2_-VA score [112].

## Management and prevention of AF through comorbidity treatment

Most patients with AF have underlying comorbidities that serve as risk factors for AF development and progression. Managing these risk factors and treating associated conditions can help prevent AF recurrence, reduce AF burden, and slow the progression to permanent AF. The ARREST-AF study [113] examined patients with AF who were obese and had other risk factors for AF. The study found that those who underwent comprehensive risk factor management, including weight loss, blood pressure control, blood sugar and lipid management, screening and treatment for sleep apnea, smoking cessation, and alcohol abstinence, had significantly lower rates of AF symptoms and recurrence compared to those who did not manage these risk factors. Additionally, patients in the risk factor management group achieved better rhythm control than those without risk factor intervention.

**Figure 12.**
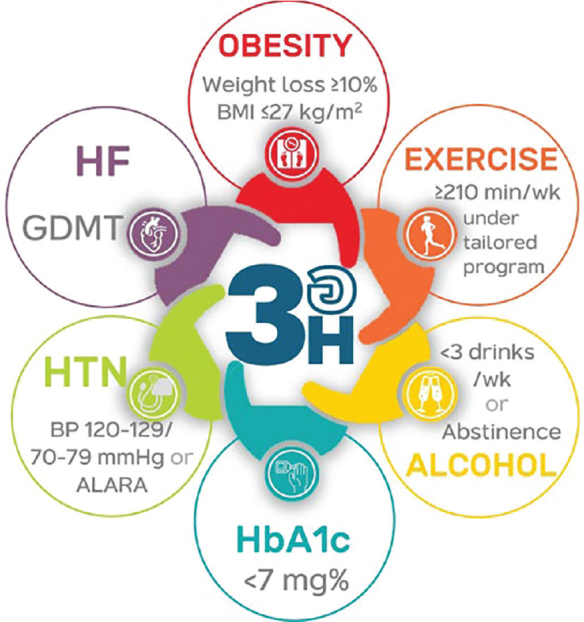
Risk factor management using the ″3อ 3H″ approach to prevent AF recurrence. AF, atrial fibrillation; BMI, body mass index; GDMT, guideline-directed management and therapy; HbA1c, hemoglobin A1c; HF, heart failure; HTN, hypertension; kg, kilogram; m^2^; square meter; min, minute.

Therefore, all AF patients should be screened for underlying risk factors and comorbidities that contribute to AF development. Clear treatment goals should be established and communicated to patients, along with comprehensive education and guidance on risk factor control and lifestyle modifications (Figure 12 and Table 18).

**Table 18.** Recommendations for the treatment and prevention of AF in all patients by managing comorbid conditions

Recommendation	Class^a^	Evidence^b^
It is recommended to identify and control risk factors and comorbid conditions that contribute to AF to reduce symptoms, prevent AF recurrence, and decrease complications associated with AF.	I	A

1 AF, atrial fibrillation.

a Class of recommendation.

b Level of evidence.

### Obesity

Obesity is a significant risk factor for incident AF and recurrence of AF after catheter ablation [114]. Weight reduction in AF patients with body mass index (BMI) ≥27 kg/m^2^ leads to in symptom improvement and a significant reduction in AF burden [115]. Additionally, the LEGACY study revealed that individuals who maintained a sustained weight loss of >10% had a significantly lower risk of AF recurrence than those with a weight loss of <3% or those experiencing weight fluctuations of >5%. Moreover, weight reduction has the potential to reverse stages of AF from persistent AF to paroxysmal AF [116]. Another study focusing on obese AF patients with BMI ≥40 kg/m2 undergoing catheter ablation found that those who underwent bariatric surgery before ablation had significantly fewer AF recurrences than those who did not have the surgery [117]. Weight loss also directly impacts cardiac structure and alters electrical conduction within the heart [118]. Notably, reductions in LA dimension, myocardial mass, and pericardial fat were observed in patients with weight reduction. Additionally, weight loss indirectly improves AF risk factors such as blood pressure, blood sugar, and lipid profiles [115] (Table 19).

**Table 19.** Recommendations for the management of AF patients with obesity or overweight

Recommendation	Class^a^	Evidence^b^
Weight reduction of at least 10% is recommended for AF patients with obesity or overweight (BMI ≥27 kg/m^2^) to improve symptoms, prevent AF recurrence, reduce AF burden, and slow disease progression.	I	B
Bariatric surgery may be considered in conjunction with comprehensive risk factor management in AF patients with a IIb BMI ≥40 kg/m^2^ who require rhythm control therapy to prevent AF recurrence.	IIb	C

1 AF, atrial fibrillation; BMI, body mass index.

a Class of recommendation.

b Level of evidence.

### Sleep apnea

Sleep apnea affects at least 20%–70% of AF patients, depending on the diagnostic methods used and the stage of AF [119]. Observational studies in AF patients who underwent rhythm control through electrical cardioversion or catheter ablation indicate that those with OSA who received treatment with continuous positive airway pressure (CPAP) experienced lower AF recurrence rates than those who did not receive treatment [120]. Although RCTs have shown that patients treated with CPAP demonstrated improved electrical conduction in the LA [121]. The benefit of CPAP in preventing AF recurrence remains unclear [122] (Table 20).

**Table 20.** Recommendations for the management of AF patients with OSA

Recommendation	Class^a^	Evidence^b^
Screening for OSA may be considered in AF patients, although the effectiveness of its treatment in preventing AF recurrence remains unclear.	IIb	B

1 AF, atrial fibrillation; OSA, obstructive sleep apnea.

a Class of recommendation.

b bLevel of evidence.

### Physical activity

Physical activity has a bidirectional relationship with AF occurrence. High-intensity physical activity exceeding 5,000 metabolic equivalents (METs)-min/week, particularly in men, has been associated with an increased risk of AF [123]. However, engaging in appropriate levels of physical activity significantly reduces AF risk. Studies indicate the optimal exercise level for reducing AF risk is 1,000–1,500 METs-min/week [124]. This level corresponds to moderate-intensity exercise (5 METs) for 200–300 min/week or vigorous-intensity exercise (7 METs) for 150–210 min/week. A RCT in AF patients demonstrated that interval training thrice weekly for 12 weeks significantly reduced symptoms, prevented AF recurrence, and improved quality of life [125]. Additionally, the intensity and duration of exercise per week influence AF occurrence. AF patients who engaged in moderate to vigorous aerobic physical activity under the tailored program for 210 min/week >6 months had significantly fewer symptoms and lower AF burden than those who performed moderate physical activity for 150 min/week [126] (Table 21).

**Table 21.** Recommendations for physical activity in patients with AF

Recommendation	Class^a^	Evidence^b^
Moderate-to-vigorous exercise training for 210 min/week is recommended for AF patients to reduce symptoms, prevent AF recurrence, improve cardiovascular and pulmonary fitness, and enhance quality of life. However, caution should be taken in elderly patients.	I	B

1 AF, atrial fibrillation.

a Class of recommendation.

b Level of evidence.

### Alcohol consumption

Consuming more than two standard drinks per day or more than five standard drinks on one occasion significantly increases the risk of AF [127]. Alcohol slows conduction and reduces the refractory period in the LA [128]. Acetaldehyde, a byproduct of alcohol metabolism, directly affects atrial muscle tissue and promotes fibrosis formation in the heart, making AF more likely to occur [129]. A RCT involving AF patients who averaged 16 standard drinks per week demonstrated that reducing alcohol intake to fewer than 3 standard drinks per week significantly decreased AF recurrence and AF burden at 6 months compared to those who did not reduce their alcohol intake [130] (Table 22).

**Table 22.** Recommendations for managing AF patients who consume alcohol

Recommendation	Class^a^	Evidence^b^
It is recommended to stop or reduce alcohol consumption to <30 g of alcohol per week (3 standard drinks per week) in AF patients to reduce AF recurrence.	I	B

1 AF, atrial fibrillation.

a Class of recommendation.

b Level of evidence.

### Diabetes mellitus

Diabetes mellitus is associated with AF and its related complications [131]. Hemoglobin A1c (HbA1c) levels are correlated with AF recurrence after catheter ablation [132]. Specifically, diabetic patients who achieved HbA1c level reduction of >10% within the 12 months before catheter ablation had significantly lower AF recurrence than those who could not [133]. Recent studies have also indicated that certain antidiabetic medications, such as metformin and sodium-glucose cotransporter-2 inhibitor (SGLT2i), can reduce AF occurrence [134]. Additionally, medications prescribed for diabetic patients with chronic kidney disease (CKD), particularly non-steroidal mineralocorticoid receptor antagonists (MRA), have also been shown to reduce AF occurrence [135] (Table 23).

**Table 23.** Recommendations for the management of AF patients with diabetes mellitus

Recommendation	Class^a^	Evidence^b^
It is recommended to control blood sugar levels, aiming for an HbA1c level of <7% in AF patients with diabetes to reduce AF burden and slow disease progression. Considerations should be made based on individual patient factors such as limited life expectancy, functional, and cognitive impairment.	I	C

1 AF, atrial fibrillation; HbA1c, hemoglobin A1c.

a Class of recommendation.

b Level of evidence.

### HTN

HTN is not only a significant risk factor for AF but is also associated with complications and cardiovascular mortality in AF patients [136]. A meta-analysis of various studies [137] found that reducing blood pressure by five mmHg in AF patients can lower the risk of cardiovascular complications by 9%. The SMAC-AF study [138] compared the effects of strict blood pressure control with standard control in AF patients undergoing catheter ablation. After 14 months of follow-up, the recurrence of AF did not significantly differ between the two groups. However, this study had several limitations, including the short duration of blood pressure control before catheter ablation, a similar reduction in blood pressure in both groups, and additional risk factors such as obesity and sleep apnea among study participants. These findings suggest that controlling blood pressure alone may not prevent AF recurrence, and a comprehensive risk management approach is necessary [113] (Table 24).

**Table 24.** Recommendations for managing AF patients with HTN

Recommendation	Class^a^	Evidence^b^
It is recommended to control blood pressure within a target range of 120–129/70–79 mmHg in AF patients with HTN, considering individual patient suitability, symptoms, and polypharmacy in elderly patients, for the benefit of preventing AF recurrence and reducing cardiovascular complications.	I	B

1 AF, atrial fibrillation; HTN, hypertension.

a Class of recommendation.

b Level of evidence.

### HF

HF and AF have a bidirectional relationship. More than half of HF patients develop new-onset AF [139]. Conversely, AF can also lead to HF. The RACE-III study [140] conducted in patients with persistent AF and mild-to-moderate HF, who were treated with electrical cardioversion and antiarrhythmic drugs, found that the group receiving comprehensive treatment for HF comorbidities, including MRA, angiotensin receptor enzyme inhibitor (ACEi)/angiotensin receptor blocker (ARB), statins, and cardiac rehabilitation had significantly lower AF recurrence rates compared to those receiving standard AF treatment alone. Beyond standard HF treatment, new guideline-directed management and therapy (GDMT) for HF, such as SGLT2i, have also been shown to reduce AF incidence in HF patients [134] (Table 25).

**Table 25.** Recommendations for the management of AF patients with HF

Recommendation	Class^a^	Evidence^b^
1. It is recommended to treat HF with appropriate guideline-directed medical therapy in AF patients with HF to reduce symptoms, decrease HF-related hospitalizations, and prevent AF recurrence.	I	B
2. It is recommended to use SGLT2i in AF patients with HF to reduce HF-related hospitalizations and cardiovascular mortality.	I	A

1 AF, atrial fibrillation; HF, heart failure; SGLT2i, sodium-glucose cotransporter-2 inhibitor.

a Class of recommendation.

b Level of evidence.

## Management of AF in specific patient groups

### Athletes

Regular moderate-intensity exercise provides cardiovascular benefits and reduces the risk of AF. However, studies in older adults have shown that the relationship between exercise intensity and AF risk follows a U-shaped pattern. Moderate-intensity exercise reduces AF incidence, whereas high-intensity exercise does not. Additionally, research indicates that high-intensity endurance exercise increases AF risk, especially in younger athletes.

Athletes with AF should always undergo evaluation for underlying causes such as structural heart disease, pre-excitation syndrome, hyperthyroidism, alcohol abuse, or illicit drug use [141]. Rate control in athletes can be challenging since beta-blockers reduce exercise capacity, while NDCCBs and digoxin may not effectively control heart rate. Furthermore, rate control medications may lead to sinus bradycardia at rest, which is common in athletes and may cause chronotropic incompetence during exercise [141].

For rhythm control, flecainide or propafenone may be considered in athletes without structural heart disease, whereas amiodarone is not recommended due to its extracardiac side effects, especially in younger athletes. In cases of paroxysmal AF, a “pill-in-the-pocket” approach with flecainide or propafenone may be used. Athletes should refrain from exercise until sinus rhythm is restored and for twice the drug’s half-life duration [91, 141]. However, flecainide or propafenone can induce AFL with 1:1 AV conduction if not combined with rate control medication. If AFL occurs, catheter ablation should be considered [142].

A prospective cohort study involving 144 athletes (mean age 50.4 years) who underwent PVI showed arrhythmia-free rates of 86%, 76%, and 56% for paroxysmal, persistent, and long-standing persistent AF, respectively, with an average of 1.4 ablation procedures per patient [143]. Athletes without recurrent AF 1 month post-PVI may resume sports participation [141]. Thus, catheter ablation is recommended for athletes with paroxysmal AF.

OAC for thromboembolism prevention in athletes should follow standard AF risk assessment. If anticoagulation is required, high-impact contact sports should be avoided to minimize bleeding risks [144] (Table 26).

**Table 26.** Recommendations for the management of AF in athletes

Recommendation	Class^a^	Evidence^b^
1. It is recommended to evaluate and treat underlying causes of AF, including structural heart disease, pre-excitation syndrome, hyperthyroidism, alcohol abuse, and illicit drug use, before resuming sports participation [141].	I	C
2. It is recommended to perform rhythm control in athletes with AF if no contraindications exist [91, 141].	I	B
3. It is recommended to perform catheter ablation in athletes with AFL to reduce the risk of AFL with 1:1 AV conduction.	I	A
4. Catheter ablation should be considered in athletes with AF [143, 145].	IIa	A
5. Athletes should not participate in sports after taking pill-in-the-pocket flecainide or propafenone until normal sinus rhythm is restored and after waiting twice the drug’s half-life duration (no more than 2 days) [91, 141].	III	B
6. Flecainide or propafenone monotherapy without rate control medication is not recommended [146].	III	C
7. Athletes receiving OACs should avoid high-impact contact sports to reduce the risk of bleeding [144].	III	C

1 AF, atrial fibrillation; AFL, atrial flutter; AV, atrioventricular; OAC, oral anticoagulant.

a Class of recommendation.

b bLevel of evidence.

### Valvular heart disease

Patients with AF frequently have coexisting valvular heart disease, particularly left-sided valvular lesions [147]. According to the Framingham Study, AF patients with RHD have a 17-fold increased risk of ischemic stroke, whereas those without RHD have a 5-fold increased risk. Additionally, a retrospective observational study conducted in Thailand found that AF patients with non-rheumatic valvular heart disease had a similar incidence of ischemic stroke compared to AF patients without valvular heart disease. However, they exhibited higher rates of GI bleeding and mortality [148]. Currently, the terms **“valvular AF”** and **“non-valvular AF”** are no longer recommended, as they can lead to confusion in thromboembolic risk assessment and patient management. Instead, it is advised to classify AF patients with valvular heart disease based on the type of OAC prescribed, into two groups.

(1) AF patients with moderate-to-severe rheumatic MS or mechanical prosthetic valve replacement require VKAs for thromboembolism prevention, regardless of their CHA_2_DS_2_-VA score. DOACs are not recommended for this group, as studies have shown that the use of rivaroxaban in AF patients with RHD (81.9% having moderate-to-severe rheumatic MS with a mitral valve area <2.0 cm^2^) resulted in higher rates of stroke, systemic embolism, myocardial infarction (MI), or vascular death compared to VKAs [45]. Additionally, the use of dabigatran in patients with aortic or mitral mechanical prosthetic valve replacements was associated with an increased incidence of thromboembolic and bleeding events [37].
(2) AF patients with other types of valvular heart disease, including mitral regurgitation, mitral valve repair, aortic stenosis, aortic regurgitation, tricuspid regurgitation, tricuspid stenosis, pulmonary regurgitation, pulmonary stenosis, bioprosthetic valve replacements, and trans-aortic valve intervention (TAVI), should be considered for either VKAs or DOACs for thromboembolism prevention based on their CHA_2_DS_2_-VA score, similar to AF patients without valvular heart disease. A retrospective observational study in Thailand found that the optimal INR range for warfarin therapy in this group should be between 2.0 and 2.49 [149]. Additionally, a meta-analysis on DOAC use in this population demonstrated that these agents have comparable efficacy and safety to those used in AF patients without valvular heart disease [150] (refer to Table 27).

**Table 27.** Recommendations for the management of AF patients with valvular heart disease

Recommendation	Class^a^	Evidence^b^
1. Warfarin is recommended for AF patients with moderate-to-severe rheumatic MS or mechanical prosthetic valve replacement for thromboembolism prevention, regardless of the CHA_2_DS_2_-VA score [151].	I	B
2. DOACs or warfarin are recommended for AF patients without moderate-to-severe rheumatic MS or mechanical prosthetic valve replacement who have an elevated stroke risk for thromboembolism prevention [150].	I	A
3. DOACs are **not recommended** for AF patients with moderate-to-severe rheumatic MS or mechanical prosthetic valve replacement [37, 45].	III	A

1 AF, atrial fibrillation; DOAC, direct oral anticoagulant; MS, mitral stenosis.

a Class of recommendation.

b Level of evidence.

### Elderly

The prevalence of AF increases with age, and aging is also associated with a higher risk of ischemic stroke, major bleeding, intracranial bleeding, HF, and mortality [152]. Additionally, elderly AF patients often have multiple comorbidities, which may increase the likelihood of adverse effects from AF treatment. Several studies have shown that early rhythm control provides benefits in elderly patients diagnosed with AF within 1 year, particularly in those aged <75 years. However, antiarrhythmic drugs should be used with caution—flecainide and propafenone should only be given after confirming the absence of structural heart disease, while amiodarone may increase the risk of adverse effects. Regarding catheter ablation using PVI, most studies have been conducted in patients with an average age of <65 years [145]. Rate control medications can be used similarly to younger AF patients, but careful consideration is required in the elderly due to potential comorbidities and side effects.

The use of OACs in elderly AF patients, particularly warfarin, requires caution, as older patients often have multiple comorbidities, increasing the risk of drug interactions with warfarin. Additionally, warfarin has a slow onset and offset, which may elevate the risk of thromboembolic or bleeding events. A study in Thailand found that the optimal INR range for patients <70 years old is 2.0–2.99, whereas for those aged >70 years, an INR range of 1.5–2.99 is recommended [50, 153]. Several studies and meta-analyses have shown that DOACs have comparable efficacy and safety to warfarin in elderly AF patients [154], with net clinical benefits observed in those aged ≥75 years [155]. However, in frail elderly AF patients (≥75 years), a study found that switching from INR-guided VKA therapy (TTR 65.3%–74%) to DOACs resulted in a higher incidence of bleeding events compared to continuing INR-guided VKA therapy [156] (Table 28).

**Table 28.** Recommendations for the management of elderly AF patients

Recommendation	Class^a^	Evidence^b^
1. Rate control therapy is recommended for elderly AF patients with a heart rate >110 bpm [70].	I	A
2. DOACs are recommended for elderly AF patients on warfarin who are unable to achieve a TTR ≥65% [48, 152, 154].	I	A
3. Consideration may be given to lowering the target INR range to 1.5–3.0 in AF patients aged ≥70 years receiving warfarin [50, 153].	IIb	B

1 AF, atrial fibrillation; DOAC, direct oral anticoagulant; INR, international normalized ratio; TTR, time in therapeutic range.

a Class of recommendation.

b Level of evidence.

Additionally, an RCT studied the use of edoxaban 15 mg once daily in elderly patients aged ≥80 years with a CHADS2 score ≥2 (mean CHA_2_DS_2_-VASc score of 4.9) who were deemed unsuitable for standard-dose OAC therapy due to CrCl of 15–30 mL/min, a history of critical organ or GI bleeding, body weight ≤45 kg, or the need for long-term non-steroidal anti-inflammatory drugs (NSAIDs) or antiplatelet therapy. The study found that edoxaban significantly reduced the risk of stroke or systemic embolism compared to placebo without increasing major bleeding, although it did elevate the risk of GI bleeding. Therefore, its use should be approached with caution.

### Wolff-Parkinson-White and preexcitation syndromes

Wolff-Parkinson-White (WPW) and preexcitation syndromes result from the presence of bridging AV working myocardium, allowing antegrade conduction from the atrium to the ventricle. The prevalence of WPW ECG patterns in the general population is 0.15%–0.25%. AF is observed in 11% of WPW syndrome patients [157]. If AF occurs in a patient with an accessory pathway with a short refractory period, it may lead to antegrade conduction, causing an extremely rapid ventricular rate of 250–300 bpm, which can result in ventricular fibrillation (VF) and cardiac arrest [157]. This condition is classified as preexcited AF. The mechanism of AF in these patients may involve atrial vulnerability, either dependent or independent of the accessory pathway, or degeneration of atrioventricular re-entrant (AVRT) tachycardia into AF.

The management of preexcited AF depends on the patient’s hemodynamic status. In cases of hemodynamic instability, electrical cardioversion is recommended [158]. For hemodynamically stable patients, intravenous procainamide or ibutilide can be used for pharmacologic cardioversion [159, 160]; however, these medications are not available in Thailand. The use of intravenous amiodarone in these patients has been reported to induce VF [161]. Additionally, AV nodal blocking agents, including adenosine, verapamil, diltiazem, beta-blockers, and digitalis, should be avoided, as they may facilitate rapid ventricular conduction and worsen the condition [162].

The most effective and appropriate treatment for preexcited AF is catheter ablation of the accessory pathway, which has an efficacy of at least 95% and a very low risk of major complications (Table 29).

**Table 29.** Recommendations for the management of preexcited AF

Recommendation	Class^a^	Evidence^b^
1. Electrical cardioversion is recommended in patients with preexcited AF [158].	I	B
2. Catheter ablation of the accessory pathway is recommended in patients with preexcited AF.	I	C
3. Intravenous adenosine, verapamil, diltiazem, beta-blockers, amiodarone, and digoxin are not recommended for acute management of preexcited AF [162–164].	III	C

1 AF, atrial fibrillation.

a aClass of recommendation.

b Level of evidence.

### Hyperthyroidism

Hyperthyroidism is a known cause of AF, and in elderly patients, AF may be the first presenting symptom of hyperthyroidism. Triiodothyronine (T3) enhances systolic depolarization and diastolic repolarization, shortens the action potential duration and refractory period of the atrial myocardium, and reduces the atrial/ventricular nodal refractory period and interatrial action potential duration. These effects create a substrate for atrial arrhythmias.

Elevated thyroid hormone levels increase the heart’s sensitivity to catecholamines [165]. Therefore, beta-blockers are the preferred choice for rate control in AF patients with hyperthyroidism, as they effectively reduce symptoms and heart rate. Non-selective beta-blockers, particularly propranolol, are more effective than selective beta-1 receptor blockers, as propranolol also inhibits the peripheral conversion of T4 to T3 [166]. Studies have shown that restoring euthyroidism leads to spontaneous conversion of AF to sinus rhythm in approximately 62% of patients, with three-fourths of them converting within 3 weeks after achieving euthyroidism. If AF persists beyond 4 months of being euthyroid, spontaneous conversion is unlikely. Therefore, if rhythm control with cardioversion is required, it is recommended to perform it from week 16 onward after the patient has achieved euthyroidism [167].

The use of antiarrhythmic drugs in AF patients with hyperthyroidism should avoid amiodarone, as it contains 37.5% iodine by weight, which can lead to amiodarone-induced thyrotoxicosis.

For OAC therapy to prevent thromboembolism in AF patients with hyperthyroidism, the decision should be based on the same risk factors as in AF patients without hyperthyroidism, as studies have shown that AF is not an independent risk factor for cerebrovascular events in hyperthyroid patients [168]. However, caution is needed when prescribing warfarin, as thyrotoxicosis increases vitamin K catabolism, leading to an elevated INR. Therefore, warfarin should be initiated at a lower dose with close INR monitoring. DOACs, on the other hand, can be used safely in these patients, as studies have found no significant differences in ischemic stroke, systemic embolism, or major bleeding between AF patients with and without hyperthyroidism when treated with DOACs (Table 30). Data supporting the discontinuation OACs in patients after hyperthyroidism become eythyroid state and AF return sinus are lacking despite the current ACC guideline recommends that OACs can be discontinued when thyroid function has returned to normal and sinus rhythm can be maintained [169]. After these patients achieves euthyroidism and sinus rhythm, whether OAC continuation should be considered based on the risk of recurrent AF, the risk of major bleeding, and patient preferences. Therefore, the net clinical benefit should be considered after shared decision between patients and physicians. Moreover, the extended monitoring for recurrent AF is required.

**Table 30.** Recommendations for the management of AF patients with hyperthyroidism

Recommendation	Class^a^	Evidence^b^
1. Non-selective beta-blockers, particularly propranolol, are recommended for rate control in AF patients with hyperthyroidism [166, 170, 171].	I	B
2. OACs are recommended for AF patients with hyperthyroidism and elevated stroke risk to prevent thromboembolism [168, 172, 173].	I	A
3. Early rhythm control should be considered in AF patients who have remained euthyroid for at least 16 weeks [167].	IIa	B

1 AF, atrial fibrillation; OAC, oral anticoagulant.

a Class of recommendation.

b Level of evidence.

### ACS/chronic coronary syndromes

The prevalence of AF in ACS patients ranges from 2% to 23% [174], occurring in both ST-elevation myocardial infarction (STEMI) and non-ST elevation acute coronary syndrome (NSTE-ACS). The combined use of antiplatelet agents and OACs remains challenging, as it may increase the risk of either stent thrombosis or an increased risk of bleeding [175]. Therefore, achieving a balance between thrombotic and bleeding risks is critical, and careful selection of both the type and duration of antiplatelet therapy and OAC therapy is essential.

**Figure 13.**
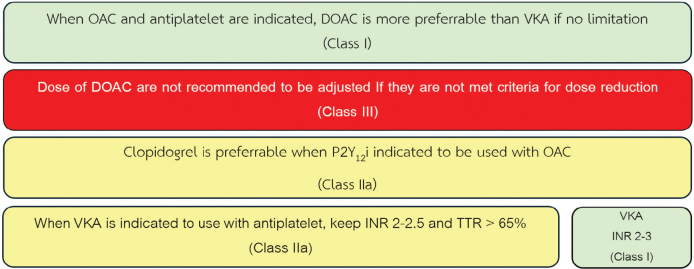
Recommendations for selecting antiplatelet drugs and OACs in patients with AF who have ACS. ACS, acute coronary syndromes; DOAC, direct oral anticoagulant; OAC, oral anticoagulant; VKA, vitamin K antagonist.

**Figure 14.**
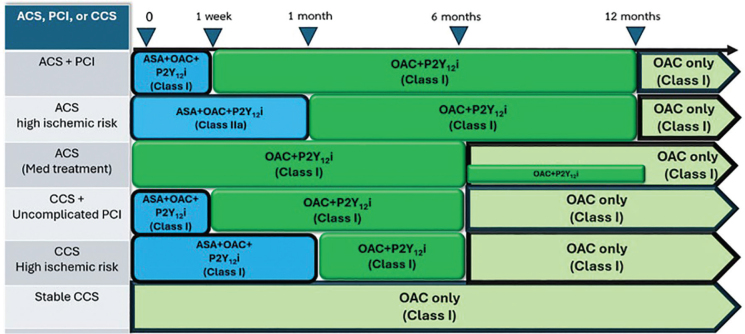
Administration of antithrombotic drugs in patients with AF who have ACS or CCS. ACS, acute coronary syndromes; AF, atrial fibrillation; ASA, aspirin; CCS, chronic coronary syndromes; OAC, oral anticoagulant; PCI, percutaneous coronary intervention; P2Y_12_i, P2Y_12_-receptor inhibitor.

Current guidelines for triple therapy in AF patients requiring antiplatelet and anticoagulant therapy are summarized in Figures 13 and 14 and Tables 31 and 32. It should be noted that although the guideline does not recommend DOAC dose reduction without appropriate criteria, considerations of low dose DOACs are possible for those with high bleeding risk. In this case, share decision making between patients and physicians is recommended.

**Table 31.** Recommendations for the use of OACs in patients with AF-ACS/CCS

Recommendation	Class^a^	Evidence^b^
1. It is recommended to consider DOAC before warfarin in patients who need to take antiplatelet drugs with OACs, if there are no contraindications or limitations to DOAC use	I	A
2. Consider rivaroxaban 15 mg once daily instead of rivaroxaban 20 mg once daily, or dabigatran 110 mg twice daily instead of dabigatran 150 mg twice daily, when coadministered with antiplatelet agents in patients who have a higher risk of bleeding than the risk of stent thrombosis or ischemic stroke.	IIa	B
3. Consider maintaining an INR level between 2 and 2.5 and a TTR level ≥65% when administering warfarin in combination with antiplatelet agents to reduce the risk of bleeding.	IIa	C

1 ACS, acute coronary syndromes; AF, atrial fibrillation; CCS, chronic coronary syndromes; DOAC, direct oral anticoagulant; INR, international normalized ratio; OAC, oral anticoagulant; TTR, time in therapeutic range.

a Class of recommendation.

b Level of evidence.

**Table 32.** Recommendations for the use of antithrombotic drugs in patients with AF undergoing PCI

Recommendation	Class^a^	Evidence^b^
1. It is recommended to administer aspirin for no longer than 1 week and to use an OAC, preferably a DOAC over warfarin, in combination with a P2Y12 inhibitor (especially clopidogrel) for up to 12 months in patients with ACS undergoing PCI, in case the risk of bleeding is higher than risk of stent thrombosis.	I	A
2. It is recommended to discontinue aspirin within the first week in patients with CCS undergoing PCI in uncomplicated cases, and to consider using a P2Y12 inhibitor in combination with an OAC for no more than 6 months to avoid the risk of bleeding.	I	A
3. Consider administering aspirin and clopidogrel in combination with an OAC for >1 week but not exceeding 1 month in patients with CCS undergoing PCI in cases risk of stent thrombosis is higher than the risk of bleeding.	IIa	B

1 ACS, acute coronary syndromes; AF, atrial fibrillation; CCS, chronic coronary syndromes; DOAC, direct oral anticoagulant; OAC, oral anticoagulant; PCI, percutaneous coronary intervention.

a Class of recommendation.

b Level of evidence.

Additionally, proton pump inhibitors (PPIs) are recommended for patients at risk of GI bleeding [176]. Antithrombotic therapy should also be adjusted based on risk assessment considering comorbid conditions in patients with ACS or chronic coronary syndromes (CCS).

### Pregnancy

AF is a common condition in pregnant women, with its prevalence increasing with maternal age, lifestyle factors, and comorbidities [177]. The rapid ventricular rate associated with AF can significantly impact maternal and fetal circulation, potentially leading to adverse outcomes and an increased risk of maternal mortality [6].

Holistic care for pregnancy with AF from a multidisciplinary team is essential to reduce symptoms, mortality, and prevent potential complications (Table 33).

**Table 33.** Recommendations for the management of AF in pregnant women [1, 2]

Recommendation	Class^a^	Evidence^b^
Unstable vital signs		
1. It is recommended to perform electrical cardioversion in pregnancy with AF, including pregnancy with preexcitation syndromes.	I	C
2. Consider performing electrical cardioversion in pregnancy with AF and concomitant HCM.	IIa	C
3. Amiodarone is not recommended owing to its adverse effects on fetus, such as goiter, abnormal fetal growth retardation, bradycardia, and the potential for preterm birth.	III	C
**Rate control**		
1. It is recommended to use beta-1 selective blockers, except atenolol, for controlling heart rate and reducing symptoms caused by AF.	I	C
2. Consider administering digitalis in case beta-blockers are ineffective or the patient cannot tolerate their side effects.	IIa	C
**Rhythm control**		
Consider administering flecainide or propafenone with AV nodal blocking agents for long-term rhythm control in pregnancy without structural heart disease when rate control agents are ineffective.	IIb	C

1 AF, atrial fibrillation; AV, atrioventricular; HCM, hypertrophic cardiomyopathy.

a Class of recommendation.

b Level of evidence.

### The use of OACs to prevent thromboembolism

The decision to use OACs to prevent thromboembolism should be a shared decision between the patient and the treating physician [1], considering the risk of thromboembolism, the benefits, and the complications of using OACs, such as in cases with rheumatic MS. It is not recommended to use DOACs due to the lack of data and evidence on their safety [178]. Guidelines for the use of anticoagulants during pregnancy and before delivery are shown in Table 34.

**Table 34.** Provides guidelines for the use of anticoagulants during pregnancy and before delivery [1]

Options for administering anticoagulants during pregnancy				
	Option 1	Option 2	Option 3	Option 4
First Trimester	Warfarin ≤5 mg/day	LMWH*	UFH	LMWH*
Second Trimester	Warfarin	Warfarin	Warfarin	LMWH*
Third Trimester until 36 weeks	Warfarin	Warfarin	Warfarin	LMWH*
**Options for administering anticoagulation after 36 weeks**				
	**Option 1**		**Option 2**	
>36 weeks	Switch warfarin to intravenous UFH.		LMWH*	
36 h prior delivery	Continue intravenous UFH		Switch LMWH to administer intravenous UFH	
4–6 h before delivery	Discontinue intravenous UFH		Discontinue intravenous UFH.	

* Administration of LMWH requires monitoring of anti-Xa levels.

1 LMWH, low molecular weight heparin; UFH, unfractionated heparin.

Pregnancy with atrial fibrillation, it is recommended to deliver according to obstetric indications.

### HCM

The prevalence of AF in patients with HCM is >25%. Rhythm control can help to reduce the likelihood of adverse changes in the LA, both anatomically and functionally to reduce ischemic stroke and cardiomyopathy [1] (Table 35).

**Table 35.** Recommendations for the management of patients with AF with HCM

Recommendation	Class^a^	Evidence^b^
Rate control		
1. It is recommended to use beta-blockers, verapamil, and diltiazem for rate control, respect to patient’s comorbidities and preference.	I	C
2. Consider performing AVN ablation in cases where heart rate cannot be controlled with antiarrhythmic drugs, the patient cannot tolerate their side effects, or AF ablation is ineffective.	IIa	B
**Rhythm control**		
1. Consider performing cardioversion or administering antiarrhythmic drugs in patients who cannot tolerate AF symptoms, respect to patients’ comorbidities and preferences.	IIa	B
2. Consider administering amiodarone after performing electrical cardioversion for rhythm control.	IIa	B
3. Consider performing catheter ablation for rhythm control in case of antiarrhythmic drugs are ineffective.	IIa	B
4. Consider surgical AF ablation in patients undergoing septal myectomy.	IIa	B
**Thromboembolism prevention**		
It is recommended to administer OACs for all patients with AF and HCM, regardless of the CHA_2_DS_2_-VA score. DOACs should be preferably considered rather than warfarin if there are no limitations for DOACs administration.	I	B

1 AF, atrial fibrillation; AVN, atrioventricular node; DOAC, direct oral anticoagulant; HCM, hypertrophic cardiomyopathy; OAC, oral anticoagulant.

a Class of recommendation.

b Level of evidence.

### Pulmonary disease

AF is commonly found in patients with chronic obstructive pulmonary disease (COPD) and is associated with increased mortality and bleeding due to the heightened risk of acute pulmonary embolism. It is recommended to use similar criteria for administering OACs as in general AF patients [1, 2] (Table 36).

**Table 36.** Recommendations for patients with AF with pulmonary disease

Recommendation	Class^a^	Evidence^b^
Consider administering cardioselective beta-blockers or NDCCB in patients with AF and COPD for rate control.	IIa	B

1 AF, atrial fibrillation; COPD, chronic obstructive pulmonary disease; NDCCB, non-dihydropyridine calcium-channel blocker.

a Class of recommendation.

b Level of evidence.

### Trigger-induced AF

Trigger-induced AF, or secondary AF, is caused by external triggers, particularly bloodstream infections, with an AF incidence of 9%–20%, which worsens the prognosis [179]. Additionally, there is a 33%–50% recurrence rate of AF in these patients [180]. Secondary AF carries a thromboembolism risk comparable to that of primary AF [173]. Therefore, treatment, especially with OACs, can be considered based on the same principles as for primary AF. It is crucial to assess the bleeding risk and engage in shared decision-making with the patient and family, particularly in specific cases (Table 37).

**Table 37.** Recommendations for the management of POAF and trigger-induced AF

Recommendation	Class^a^	Evidence^b^
POAF prevention		
1. Short-term amiodarone should be considered in patients undergoing cardiac or pulmonary surgery with a high risk of developing AF.	IIa	B
**Management of POAF and trigger-induced AF**		
1. Heart rate control should be maintained <110 bpm using medications such as beta-blockers or NDCCBs, provided there are no contraindications.	I	A
2. Consideration of additional rhythm management with antiarrhythmic medications is recommended based on the patient’s symptoms, hemodynamic stability, and the clinical judgment of the treating physicians [181, 182].	I	A
3. Electrical cardioversion combined with antiarrhythmic drugs is recommended for patients with hemodynamic instability, if AF occurs within 48 h or no thrombus is found in the atrium.	I	B
4. Long-term OACs should be considered in trigger-induced AF patients at high risk of ischemic stroke and thromboembolism.	IIa	C
5. Follow-up evaluation at 30–60 days should be considered to assess the need for rhythm control after appropriate use of OACs [173].	IIa	C

1 AF, atrial fibrillation; NDCCB, non-dihydropyridine calcium-channel blocker; OAC, oral anticoagulant; POAF, post-operative atrial fibrillation.

a Class of recommendation.

b Level of evidence.

### Post-operative atrial fibrillation

Post-operative atrial fibrillation (POAF) can occur in 30%–50% of patients undergoing cardiac surgery and 5%–30% of those undergoing other types of surgery [183]. Risk factors include both pre-existing conditions and triggers from bodily changes during and after surgery, as well as complications that arise, all of which contribute to the development of this condition [184]. Although most cases resolve once the triggers are removed, the recurrence rate of AF in the 5-year period for these patients increases by 4–5 times [185]. POAF also raises the risk of ischemic stroke, myocardial ischemia, HF, and mortality [186].

The use of amiodarone to prevent AF in patients undergoing cardiac surgery has been found to be equally effective as beta-blockers, with fewer side effects, such as a lower incidence of ischemic stroke [187]. Therefore, Short-term amiodarone should be considered in patients undergoing cardiac or pulmonary surgery with a high risk of developing AF [188–190] (Table 37). The definition of high risk for developing post-operative AF was based on the ESC guideline [2] which included at least one of the following: (1) clinical risk factors (such as age at least 65 years, history of AF, COPD, diabetes), (2) surgical risk factors [such as coronary artery bypass grafting (CABG), mitral valve surgery, lobectomy or pneumonectomy] (3) biomarkers and imaging [such as elevated N-terminal pro-B-type natriuretic peptide (NT-proBNP), enlarged LA], and (4) risk scores (such as CHA_2_DS_2_-VASc score). While beta-blockers are effective in reducing AF occurrence and myocardial ischemia, studies have shown that administering beta-blockers before surgery increases the risk of death and ischemic stroke [191]. Thus, beta-blockers are not recommended for AF prevention in surgical patients.

Posterior pericardiotomy is a procedure that helps relieve pericardial fluid accumulation. According to the PALACS study, this procedure significantly reduces the incidence of AF in patients undergoing cardiac surgery [192], and it should be considered as part of the surgical approach. Other treatments, such as the use of colchicine, corticosteroids, magnesium, and sotalol, as well as procedures like botulinum toxin injection into epicardial fat, have no supporting evidence to reduce AF incidence in surgical patients [193].

The use of OACs, especially in patients with thromboembolism risk, can reduce the incidence of thromboembolic events [194]. However, the increased risk of bleeding should also be considered. Therefore, treatment should be initiated when the patient’s bleeding risk is sufficiently low.

### Cancer

Cancer patients are at higher risk of developing AF, with an incidence ranging from 2% to 28% [195]. In addition to pre-existing risk factors for atrial arrhythmias, factors such as cancer type, treatments (e.g., surgery, chemotherapy, or radiotherapy) can also contribute to the development of AF [196]. Therefore, monitoring and assessment of patients undergoing treatments that increase their risk should be performed periodically (Table 38).

**Table 38.** Cancer treatments increasing the risk of AF [197]

Cancer treatments	Cancer treatments	
	General Common Incidence: Incidence: 1%–10%	>10%
**Anthracyclines**		
Doxorubicin, epirubicin, idarubicin, mitoxantrone		X
**Antimetabolites**		
Clofarabine combined with cytarabine	X	X
5-FU	X	
Cepecitabine		
Gemcitabine	X	
**Alkylating agents**		
Cyclophosphamide	X	X
Melphalan + stem cell transplantation		
**Immunomodulatory drugs**		
Lenalidomide	X	
Interleukin-2		
**TKIs**	X	
Ibrutinib (BTK inhibitors)	X	X
Acalbrutinib (second-generation BTK	X	
inhibitors)	X	
Zanubrutinib (second-generation BTK	X	
inhibitors)	X	
Ponatinib (BCR-ABL TKI) and other TKIs (e.g., trametinib, osimertinib, nilotinib, ribociclib)	X	
**VEGF inhibitor**		
Sorafenib in combination with 5-FU		
**BRAF inhibitor**		
Vemurafenib		
**CAR-T**		
Tisagenlecleucel	X	
Axicabtagene ciloleucel	X	
**Monoclonal antibodies**		
Rituximab	X	

1 5-FU, 5-fluorouracil; AF, atrial fibrillation; BCR-ABL, breakpoint cluster region-Abelson oncogene locus; BTK, Bruton tyrosine kinase; CAR-T, chimeric antigen receptor T cell; TKIs, tyrosine kinase inhibitors; VEGF, vascular endothelial growth factor.

A key consideration in the treatment of cancer patients is evaluating the benefits of OACs in preventing thromboembolism against the risks of major bleeding and mortality associated with these medications, necessitating close monitoring of patient symptoms and risk assessment. In this group, DOACs have demonstrated greater efficacy and safety in preventing thromboembolism compared to VKAs, similar to other patient populations. Additionally, an important factor to consider is potential drug interactions between OACs and cancer therapies, particularly tyrosine kinase inhibitors (TKIs) such as imatinib, crizotinib, and sunitinib, as well as hormonal therapy agents like abiraterone and enzalutamide. Therefore, careful evaluation of drug interactions is necessary to ensure appropriate anticoagulant selection for each patient.

### CKD

AF is increasingly prevalent in patients with CKD and is associated with a 67% increased risk of kidney failure in CKD patients diagnosed with AF [198]. The management of AF in CKD patients, including catheter ablation and the use of antiarrhythmic drugs, is often limited by safety concerns identified in multiple studies. Among antiarrhythmic drugs, amiodarone remains the primary option due to its established safety profile and the lack of need for dose adjustment [1]. Given the limitations of pharmacological therapy, catheter ablation may be a viable treatment option for these patients; however, careful consideration should be given to the risks associated with fluid overload during the procedure.

Since the kidneys play a crucial role in the pharmacokinetics of drugs, the dosing of OACs in patients with kidney disease is adjusted based on renal clearance, as shown in Table 39. In patients with severe renal impairment, defined as a CrCl of <15 mL/min or those undergoing dialysis, current data on the efficacy of OACs remain insufficient to support routine use. Anticoagulants do not effectively reduce thromboembolism risk and are associated with an increased incidence of major bleeding compared to no anticoagulation [199]. Data on DOAC use in this population from retrospective cohort studies and small RCTs suggest comparable efficacy and safety to VKAs, particularly with rivaroxaban [199] and apixaban at a dose of 2.5 mg twice daily [200]. However, as clinical evidence in this patient group remains inconclusive, the decision to use OACs should be carefully discussed with the patient and their family on a case-by-case basis.

**Table 39.** Dosage of OACs in AF patients with CKD and cirrhosis [93]

Drug Class	VKA	Direct thrombin inhibitor	Factor Xa inhibitor		
Drug name	Warfarin	Dabigatran	Rivaroxaban	Apixaban	Edoxaban
CKD					
CrCl 50–90 mL/min	INR 2–3	150/110 mg bid	20 mg od	5/2.5 mg bid^a^	60 mg od
CrCl 30–50 mL/min	INR 2–3	150/110 mg bid	15 mg od	5/2.5 mg bid^a^	30 mg od
CrCl 15–30 mL/min	INR 2–3	Avoid use	15 mg od	2.5 mg bid	30 mg od
CrCl <15 mL/min or dialysis	**Insufficient data to recommend use^b^**				
**Liver disease**					
Child-Pugh A (Mild)	INR 2–3	No dose adjustment required	No dose adjustment required	No dose adjustment required	No dose adjustment required
Child-Pugh B (Moderate)	INR 2–3	Use with caution	Avoid use	Use with caution	Use with caution
Child-Pugh C (Severe)	INR 2–3	Avoid use	Avoid use	Avoid use	Avoid use
Class of recommendation	I	IIa	IIb	III	
a. Adjust to a dose of 2.5 mg if at least two of the following apply: age ≥80 years, weight ≤60 kg, creatinine >1.5 mg/dL					
b. Depends on shared decision-making between the physician and the patient.					

1 AF, atrial fibrillation; CKD, chronic kidney disease; CrCl, creatinine clearance; min, minute; INR, international normalized ratio; OAC, oral anticoagulant; VKA, vitamin K antagonist.

The PRAGUE-17 study [201] demonstrated that LAAO was non-inferior to DOACs in reducing primary composite endpoints, including the incidence of ischemic stroke, major bleeding, and procedural complications. Therefore, LAAO may be considered as an alternative for this patient group. However, a detailed and thorough discussion with the patient and their family is essential, taking into account the patient’s preferences and expectations before making a treatment decision.

### Liver cirrhosis

The liver is essential for waste elimination and plays a crucial role in the clearance of OACs. As a result, the primary studies on DOACs excluded patients with liver disease, who are at a higher risk for major bleeding. However, real-world data and meta-analyses of patients with liver disease have shown that DOACs can reduce major bleeding and intracranial hemorrhage compared to VKAs [202]. Nonetheless, caution is required when using DOACs in patients with cirrhosis. Specifically, if a patient with cirrhosis has a Child-Pugh score of B, dabigatran, apixaban, and edoxaban should be used with caution, while rivaroxaban should be avoided. In patients with cirrhosis and a Child-Pugh score of C, all DOACs should be avoided, as shown in Table 39. Additionally, before initiating OACs in cirrhosis patients, potential complications, such as esophageal varices, should be assessed and treated to reduce the risk of bleeding in this group.

## Key messages from Thai AF guidelines 2025

1. A patient-centered, comprehensive approach based on the “ไม่เอา 3 อ” strategy is crucial for AF management. The “ไม่เอา 3 อ” strategy which read “Mai Aou Sam Or” means ”no 3 ”no 3 อ”. These “อ” are the initial letter for Thai words for stroke, symptoms, and obesity
  1.1. “ไม่เอาอัมพฤกษ์-อัมพาต” means “No Stroke”—Stroke prevention through anticoagulation therapy.
  1.2. “ไม่เอาอาการ” means “No Symptoms”—Symptom control via rate or rhythm management.
  1.3. “ไม่เอาอ้วนและอื่นฯ” means “No Obesity and Other Factors”—Management of underlying conditions.
2. Stroke and thromboembolism prevention
  2.1. OAC is recommended in individuals with an annual risk >2% (CHA_2_DS_2_-VA score ≥2, HCM, moderate to severe MS, and mechanical heart valve) and to be considered for those with an annual risk >1and <2% (CHA_2_DS_2_-VA = 1).
  2.2. Bleeding risk should be considered, but high bleeding risk is not a contraindication for anticoagulants. Addressing modifiable bleeding risk factors as appropriate to lessen the chance of bleeding complications.
  2.3. Choice of anticoagulation: Once a decision has been made to use OAC, DOACs are recommended as the first-line treatment in AF patients without moderate-to-severe rheumatic MS or mechanical heart valves, as appropriate for individual healthcare settings. It is recommended to use warfarin in AF patients with moderate-to-severe rheumatic MS or mechanical heart valves.
  2.4. Target INR of 2.0–3.0 is recommended in AF patients receiving warfarin. Lowering the target INR range to 1.5–3.0 may be considered in AF patients aged ≥70 years, or 2.0–2.5 with concomitant use of antiplatelets.
  2.5. Antiplatelet therapy is not recommended for preventing ischemic stroke and thromboembolism in AF patients
3. Symptoms control
  3.1. Rate control heart rate control with medication is an essential foundational treatment to reduce symptoms from a rapid heart rate and prevent long-term complications. Beta-blockers, NDCCBs (verapamil or diltiazem), or digoxin should be used for rate control in patients with AF and LVEF >40%. For those with LVEF ≤40% betablockers or digoxin should be used.
  3.2. Rhythm control, as appropriate for the individual healthcare setting, should be considered for a patient who has significant symptoms of AF despite rate control and in patient with early AF.
  3.2.1 Antiarrhythmic drugs
    Flecainide, propafenone, or dronedarone are the first-line choice in patients without structural heart disease. Drone-darone for patients with other heart diseases without severe HF. Amiodarone is recommended for patients with severe HF or in cases where other antiarrhythmic drugs cannot be used.
  3.2.2 Catheter ablation
    Catheter ablation is recommended for patients with symptomatic paroxysmal or persistent AF despite the use of antiarrhythmic drugs, patients with AF and HFrEF, and may be considered as a first-line treatment in selected cases.
  3.2.3 Cardioversion
    To restore sinus rhythm using cardioversion, pharmacologically or electrically, it’s important to prescribe anticoagulation before and after cardioversion, according to each patient’s risk profile.
  3.2.4 Rhythm control is not a substitute for anticoagulation. Long-term continuation of OAC is recommended regardless of the success of rhythm control
4. Risk factors and co-morbidity management
  It is recommended to identify and control risk factors and comorbid conditions that contribute to AF to reduce symptoms, prevent AF recurrence, and decrease complications associated with AF. An acronym, similar to “Iman 3 a”, is used “3 a 3 h” which refers to
  4.1. “อ้วน” for obesity, an individual with a maintained BMI >27kg/m^2^ should lose 10% of the patient’s body weight.
  4.2. “ออกกำลังกาย” for exercise 210 min/week.
  4.3. “แอลกอฮอล์” for alcohol abstinent or less than 3 serving per week.
  4.4. HgA1c: to treat DM with HbA1c goal lower than 7 mg%.
  4.5. HTN: to control HTN down to 120–129/70–79 mmHg or as low as reasonably achievable.
  4.6. HF: to treat HF with appropriate guideline-directed medical therapy in AF patients with HF
5. AFL is managed similarly to AF
6. Screening for AF is recommended in high-risk populations for thromboembolic complications using pulse palpation or commercially available devices.
7. Specific patient population recommendations are provided, including the following
  7.1. Athlete
  7.2. Valvular heart disease
  7.3. Elderly
  7.4. Pre-excitation syndrome
  7.5. Hyperthyroidism
  7.6. Acute and CCS
  7.7. Pregnancy
  7.8. HCM
  7.9. Pulmonary disease
  7.10. Trigger-induced AF
  7.11. Post-operative AF
  7.12. Cancer
  7.13. CKD
  7.14. Cirrhosis
